# A kinetic-based sigmoidal model for the polymerase chain reaction and its application to high-capacity absolute quantitative real-time PCR

**DOI:** 10.1186/1472-6750-8-47

**Published:** 2008-05-08

**Authors:** Robert G Rutledge, Don Stewart

**Affiliations:** 1 Natural Resources Canada, Canadian Forest Service, 1055 du P.E.P.S., Quebec, Quebec G1V 4C7, Canada

## Abstract

**Background:**

Based upon defining a common reference point, current real-time quantitative PCR technologies compare relative differences in amplification profile position. As such, absolute quantification requires construction of target-specific standard curves that are highly resource intensive and prone to introducing quantitative errors. Sigmoidal modeling using nonlinear regression has previously demonstrated that absolute quantification can be accomplished without standard curves; however, quantitative errors caused by distortions within the plateau phase have impeded effective implementation of this alternative approach.

**Results:**

Recognition that amplification rate is linearly correlated to amplicon quantity led to the derivation of two sigmoid functions that allow target quantification via linear regression analysis. In addition to circumventing quantitative errors produced by plateau distortions, this approach allows the amplification efficiency within individual amplification reactions to be determined. Absolute quantification is accomplished by first converting individual fluorescence readings into target quantity expressed in fluorescence units, followed by conversion into the number of target molecules via optical calibration. Founded upon expressing reaction fluorescence in relation to amplicon DNA mass, a seminal element of this study was to implement optical calibration using lambda gDNA as a universal quantitative standard. Not only does this eliminate the need to prepare target-specific quantitative standards, it relegates establishment of quantitative scale to a single, highly defined entity. The quantitative competency of this approach was assessed by exploiting "limiting dilution assay" for absolute quantification, which provided an independent gold standard from which to verify quantitative accuracy. This yielded substantive corroborating evidence that absolute accuracies of ± 25% can be routinely achieved. Comparison with the LinReg and Miner automated qPCR data processing packages further demonstrated the superior performance of this kinetic-based methodology.

**Conclusion:**

Called "linear regression of efficiency" or LRE, this novel kinetic approach confers the ability to conduct high-capacity absolute quantification with unprecedented quality control capabilities. The computational simplicity and recursive nature of LRE quantification also makes it amenable to software implementation, as demonstrated by a prototypic Java program that automates data analysis. This in turn introduces the prospect of conducting absolute quantification with little additional effort beyond that required for the preparation of the amplification reactions.

## Background

Of the many attributes of real-time quantitative PCR (qPCR), the ability to conduct absolute quantification is arguably the most significant. From a technical perspective, absolute quantification allows assay performance to be precisely defined, from which sensitivity, effective quantitative range and quantitative accuracy can be expressed in absolute terms. From an application perspective, assessing biological significance within the context of absolute number of target molecules can enhance the utility of most, if not all, quantitative assays. Many prominent examples come from biomedical diagnostics where absolute quantification can have direct clinical relevancy, as is evident for monitoring viral load and residual disease. Although more general applications such as environmental screening and pathogen detection would also benefit greatly, it is the application of absolute qPCR to gene expression profiling that holds some of the most substantive implications.

Historically, real-time qPCR has largely been relegated to a supportive role in large-scale gene expression studies, most frequently used for the verification of DNA microarray datasets [1-3]. Nevertheless, absolute qPCR has the potential to extend gene expression analysis beyond what is possible with microarray analysis, based upon the innate capability to overcome two of the greatest limitations of microarray quantification, which are limited sensitivity and lack of absolute scale [4]. Some of the most illustrative examples come from the application of microarrays to clinical research and diagnostics. Founded upon the expectation that gene expression analysis can be used as a diagnostic tool to both predict and follow therapeutic outcomes [4,5], many studies have reported that effective diagnoses can be achieved with relatively small groups of biomarker transcripts, numbering between 10 and 100 [6,7], a range that is potentially within the capacity of absolute qPCR technologies.

Development of diagnostic assays for disease prediction could thus exploit the reduced technical complexity, speed of analysis, sensitivity and substantively greater resolution provided by real-time qPCR, as compared with microarray analysis [8]. Indeed, absolute quantification could increase the efficacy of any gene expression profiling initiative, irrespective of the experimental context. Nevertheless, a prominent inadequacy of current real-time qPCR technologies is the limited capacity for conducting absolute quantification, due to reliance on target-specific standard curves [9]. Not only does this necessitate preparation of a quantified standard for each target under investigation, the technical difficulties and extensive resources required for standard curve construction present considerable challenges for conducting absolute quantification, even for a modest number of targets.

A number of studies have attempted to overcome the innate limitations of standard curves by analyzing the fluorescence readings generated by individual amplification reactions. These include determining amplification efficiency through the application of exponential mathematics to the log-linear region, using either linear regression [10-13] or nonlinear regression [14,15]. Attempts to model the entire amplification profile have included sigmoidal modeling using nonlinear regression [16-20], in addition to the application of various biochemical-based models [21-23], and other forms of mathematical modeling [24-26]. Demonstration that absolute quantification can be achieved by combining optical calibration with sigmoidal modeling (a method called "sigmoidal curve fitting" or SCF [18]) has led several groups to evaluate this approach as an alternative to standard curve-based quantification [27-33]. Unfortunately, effective implementation of SCF has been impeded by errors produced by distortions within the plateau phase, which can severely compromise the accuracy of SCF-based quantification [18,31-33].

The study presented here extends SCF quantification by adapting the sigmoid function upon which SCF is based, to directly model PCR amplification without the need to conduct nonlinear regression. Based upon a linear relationship between amplification rate and amplicon quantity, this allows target quantification to be conducted using linear regression analysis. In addition to eliminating errors produced by plateau phase anomalies, this provided the foundation for development of a new quantitative paradigm that does not require standard curves and is able to resolve quantitative differences on the order of 0.5 fold, while providing unprecedented quality control capabilities. A prototypic Java program which automates implementation of this kinetic-based methodology further illustrates the potential to develop high-throughput applications, in addition to providing a visual illustration of the underlying principles.

## Results

### Amplification efficiency is dynamic and is coupled to amplicon DNA quantity

New insights into the dynamics of PCR amplification have been gained through the application of sigmoidal modeling [11,18], in which nonlinear regression analysis is used to fit real-time SYBR Green I fluorescence readings to the sigmoid function:

(1) $$F_{C}=\frac{F_{\max}}{1+{\text{e}}^{-\left(\frac{C-C_{1/2}}{k}\right)}}+F_{b}$$

where *F*_*C *_is the reaction fluorescence at cycle *C *and is proportional to the mass of amplicon DNA present in the reaction, *F*_*max *_is the maximal reaction fluorescence that defines the end point of the amplification process, referred to as the plateau phase, *C*_1/2 _is the fractional cycle at which reaction fluorescence reaches half of *F*_*max*_, *k *is related to the slope of the curve, and *F*_*b *_is the fluorescence background. Despite its apparent novelty, this equation is in fact a simple derivative of the classic Boltzmann four-parametric sigmoid function that is commonly used to model sigmoidal datasets.

Notwithstanding the complexities of conducting nonlinear regression analysis, the remarkable precision that can be achieved is indicative of the potential for sigmoidal modeling to fundamentally revolutionize real-time qPCR [18]. An essential insight into this potential comes from examination of PCR amplification kinetics, as described by a second sigmoid function [11]:

(2) $$E_{C}=\frac{1+{\text{e}}^{-\left(\frac{C-1-C_{1/2}}{k}\right)}}{1+{\text{e}}^{-\left(\frac{C-C_{1/2}}{k}\right)}}-1$$

where *E*_*C *_is the amplification efficiency at cycle *C*, also referred to as "cycle efficiency" [18]. Under a sigmoidal model, amplification rate is maximal at the onset of thermocycling, but progressively decreases such that each cycle has a unique amplification efficiency, with entry into the plateau phase occurring as amplification efficiency approaches zero.

**Figure 1:**
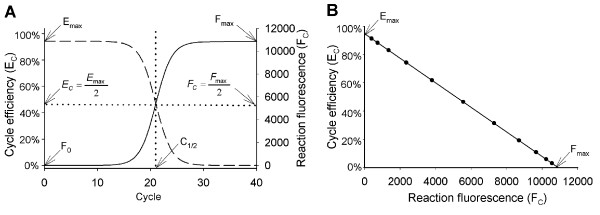
**Sigmoidal modeling of PCR amplification reveals symmetry between amplicon accumulation and loss in amplification efficiency**. **(A) **Plots of the sigmoid functions describing amplicon accumulation (equation 1, solid line) and cycle efficiency (equation 2, dashed line) illustrate a symmetrical relationship, where C_1/2 _defines the fractional cycle when both reach half of their respective maxima. F_0 _is target quantity expressed in fluorescence units, E_max _is the maximal amplification efficiency and F_max _is the maximal reaction fluorescence. **(B) **Plotting cycle efficiency against reaction fluorescence produces a line, predicting that the progressive loss in cycle efficiency is linearly coupled to amplicon accumulation. This generates a linear representation of PCR amplification (referred to as the "LRE plot"), where the Y intercept defines the maximal amplification efficiency (E_max_) when F_C _= 0 and the X intercept defines the end-point or plateau phase (F_max_) when E_C _= 0, with the slope defining the rate of loss in cycle efficiency (ΔE) in relation to amplicon DNA quantity as reflected by reaction fluorescence.

The principle of this and other insights can be illustrated by comparing plots generated with equations 1 and 2, the most notable being the striking symmetry between amplicon DNA accumulation and reduction in cycle efficiency (Figure 1A). Importantly, this implies that an association exists between amplicon DNA quantity and amplification efficiency, a contention supported by the mathematical prediction of a linear relationship between reaction fluorescence and cycle efficiency (Figure 1B).

Recognition of this linear relationship not only impacts the practicalities of modeling PCR amplification, but also has unparalleled implications for how real-time qPCR can be implemented. Central to this is the prediction that the dynamics of PCR amplification can be described by a linear equation, defined here as:

(3) *E*_*c *_= Δ*E *× *F*_*c *_+ *E*_max_

where the slope defines the rate of loss in cycle efficiency (*ΔE*) and the intercept defines the maximal amplification efficiency (*E*_*max*_) when *F*_*C *_= 0 (Figure 1B). It is also evident that as PCR amplification enters the plateau phase, reaction fluorescence approaches a maximum (*F*_*max*_) as *E*_*C *_approaches zero, such that equation 3 becomes:

0 = Δ*E *× *F*_max _+ *E*_max_

so that:

(4) $$F_{\max}=\frac{E_{\max}}{-\Delta E}$$

In addition to greatly simplifying the mathematics describing amplification kinetics, of practical significance is the ability to obtain estimates of *E*_*max *_and *ΔE *via linear regression analysis, utilizing the fluorescence readings produced by individual PCR reactions. Termed "linear regression of efficiency" or LRE, this approach not only generates a linear representation of PCR amplification, but as described in later sections, allows target quantity to be determined directly from individual fluorescence readings. An important qualification, however, is the extent to which experimental data comply with these mathematical predictions.

### Conformity of real-time amplification profiles generated with SYBR Green I

A fundamental approach to analyzing the kinetics of PCR amplification is based upon defining amplification efficiency as the relative increase in amplicon DNA over a single cycle:

(5) $$E_{C}=\frac{F_{C}}{F_{C-1}}-1$$

where *F*_*C*-1 _is the reaction fluorescence of the preceding cycle. This provides an estimate of cycle efficiency from which kinetic analysis can be conducted without having to resort to nonlinear regression analysis. It should be noted that Alvarez et al. (2007) [19] have utilized a similar approach for determining *E*_*max *_(which they refer to as the "intrinsic" amplification efficiency), although they concluded that a two-parametric sigmoid function was superior to the linear model upon which LRE is based. Nevertheless, the validity of equation 5 is dependent on the assumption that reaction fluorescence remains proportional to amplicon DNA quantity throughout the amplification process. While this may be most evident for fluorescent dyes such as SYBR Green I, other detection chemistries may not conform well to this assumption (e.g. see Swillens et al. (2004) [34]). In fact SYBR Green I detection may not be free of anomalies, particularly at high amplicon DNA quantities. This presumption is based upon the low quantities of SYBR Green I present in commercial real-time PCR formulations (estimated to be 0.1–0.2X), made necessary by the inhibitory nature of SYBR Green I. This in turn could distort the apparent cycle efficiency as predicted by equation 5, potentially invalidating, or at least distorting, LRE-based estimates of *E*_*max *_and *ΔE*.

A major goal of this study was therefore to evaluate the efficacy of sigmoidal modeling for conducting absolute quantification, with the explicit objective of validating the quantitative competency of a LRE-based methodology; that is, beyond simply generating target quantities that lack context. Central to this initiative was the utilization of bacteriophage lambda genomic DNA (lambda gDNA) as a highly defined, commercially available quantitative standard. Not only does this approach make much of this work amenable to experimental replication, it serves to illustrate some of the exceptional attributes of employing lambda gDNA as a universal quantitative standard. Paramount is the ability to establish quantitative scale, which is essential to conducting absolute quantification. As will be illustrated in later sections, this is also requisite to eliminating the need for standard curves.

Notwithstanding the potential utility of LRE-based quantification, an essential starting point was to examine the potential distortion of reaction fluorescence generated by low quantities of SYBR Green I. It was initially surmised that increasing SYBR Green I quantity would be informative; however, the sensitivity of QuantiTect (a *T. aquaticus *DNA polymerase (Taq) based formulation) to SYBR Green I inhibition precluded the ability to apply anything but a moderate increase in SYBR Green I quantity. Subsequently, two alternative non-Taq formulations were evaluated, based on the speculation that they could be more resilient to higher quantities of SYBR Green I. These were DyNAmo, formulated with an engineered *T. brockianus *DNA polymerase fused to a non-specific DNA-binding region, and FullVelocity, formulated with an unspecified archaeal DNA polymerase.

**Figure 2:**
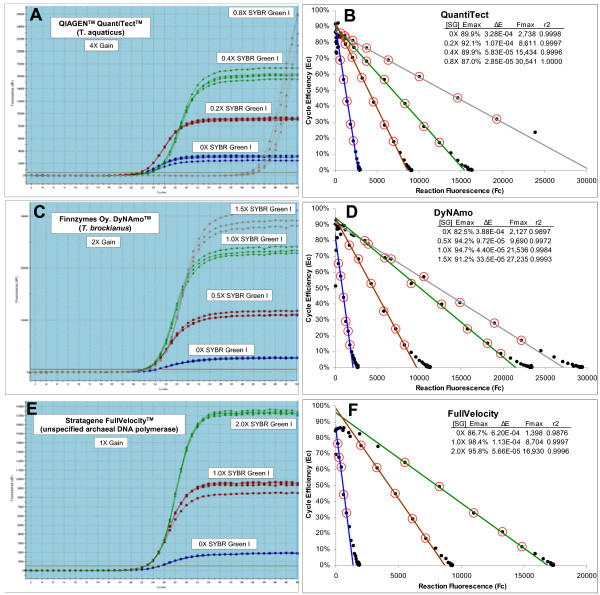
**Assessing the impact of SYBR Green I quantity on real-time amplification profiles**. Replicate amplification reactions were prepared using three commercial enzyme formulations supplemented with increasing amounts of SYBR Green I. Each amplification reaction contained 100 femtograms of lambda gDNA (1,876 genomes) and 500 μM of the primers K7B and K12. The gain setting for each run was adjusted to ensure that reaction fluorescence remained below the saturation level of the photomultiplier tube (about 40,000 FU). **(A)**, **(C) **and **(E) **Screen shots of amplification profiles generated by the Stratagene MxPro qPCR software. This reveals that fluorescence intensity is dependent on SYBR Green I quantity, reflected by the large increase in the height of the amplification profiles as SYBR Green I quantity is increased. **(B)**, **(D) **and **(F) **LRE plot of each respective amplification profile. This reveals a linear domain corresponding to the central region of each amplification profile, confirming the mathematical prediction that amplification efficiency is linearly coupled to amplicon quantity. A consecutive group of points was selected (designated by red circles) for linear regression analysis (referred to as "LRE analysis"), generating estimates for *E*_*max *_(intercept) and *ΔE *(slope), from which *F*_*max *_was calculated using equation 4 (see Figure 1 for additional details). Details as to how the boundaries of the linear region were determined are described later in the study. **[SG]**: quantity of supplementary SYBR Green I, **r2**: linear regression correlation coefficient.

As summarized in Figure 2, three series of replicate amplification reactions supplemented with progressively greater quantities of SYBR Green I demonstrate, based on similarity in amplification profile position, that addition of 1-2X SYBR Green I was marginally inhibitory to both DyNAmo and FullVelocity. In contrast, QuantiTect was greatly impacted by addition of 0.8X SYBR Green I, as reflected by the large shift in position, and extensive scattering, of the replicate amplification profiles (Figure 2A). More significant, however, is the substantive increase in F_max _produced by increasing SYBR Green I, establishing that reaction fluorescence intensity is dependent on SYBR Green I quantity. Notwithstanding this dependency, increasing SYBR Green I quantity had no apparent impact on the general sigmoidal shape of the amplification profiles. The amplification profiles produced by DyNAmo and FullVelocity further indicate that the sigmoidal shape of SYBR Green I amplification profiles is not unique to Taq.

The sigmoidal character of these amplification profiles was further substantiated by LRE analysis, which revealed a linear domain corresponding to the central region of each amplification profile (Figure 2B, D and 2F). These plots further reveal that the fluorescence readings generated by later cycles do not conform well to the LRE model, as reflected by the "spilling" of points off of the LRE line. Importantly, this is consistent with what was previously observed during development of the SCF method, where anomalies associated with the plateau phase were common, and significantly distorted nonlinear regression analyses. This subsequently required exclusion of the plateau cycles from the nonlinear regression, based upon a somewhat esoteric method for selecting a "cutoff cycle" [18]. Nevertheless, as is apparent in the LRE plots presented in Figure 2, a kinetic-based approach provides the ability to identify anomalous fluorescence readings based on loss of conformity with the LRE model.

Thus, while testing of the quantitative capabilities of LRE analysis requires additional tools, this initial assessment does provide substantive evidence that SYBR Green I real-time profiles can conform well to that predicted by sigmoidal modeling. Furthermore, these results suggest that despite the apparent complexities of the classic Boltzmann sigmoid function, it should be possible to develop a sigmoidal model for the polymerase chain reaction, derived from the two kinetic parameters predicted by the LRE model to govern PCR amplification.

### Derivation of a kinetic-based sigmoid model for the polymerase chain reaction

Conformity of PCR amplification to the classic Boltzmann sigmoid function (equation 1) poses the question as to how *C*_1/2_, *k *and *F*_*max *_relate to *ΔE *and *E*_*max*_. Although equation 4 predicts that *F*_*max *_is defined by the ratio of *E*_*max *_to *ΔE*, it is less clear how *k *and *C*_1/2 _relate. As summarized in additional file 1, *C*_1/2 _and *k *can be eliminated through a series of rearrangements and substitutions, producing two functions that allow modeling of PCR amplification based solely on *ΔE *and *E*_*max*_. On the assumption that the fluorescence background (F_b_) is zero, these are:

(6) $$F_{0}=\frac{F_{\max}}{1+\left(\frac{F_{max}}{F_{C}}-1\right){\left(E_{\max}+1\right)}^{C}}$$

(7) $$F_{C}=\frac{F_{\max}}{1+\left(\frac{F_{max}}{F_{0}}-1\right){\left(E_{\max}+1\right)}^{-C}}$$

Thus, once values for *ΔE *and *E*_*max *_have been obtained via LRE analysis, equation 6 can be used to convert individual F_C _readings into target quantity expressed in fluorescence units (F_0_). Target quantification is then based on averaging the F_0 _values derived from the cycles used in LRE analysis, followed by conversion into the number of target molecules via optical calibration. Furthermore, once an average F_0 _value has been obtained, the corresponding amplification profile can be modeled by using equation 7 to calculate predicted F_C _values for each cycle. As illustrated in the next section, this series of computations is capable of modeling PCR amplification to a very high degree of precision, and without the need to conduct nonlinear regression analysis.

It should noted that Chervoneva et al. (2007) [20] have recently described modeling of real-time PCR using a logistic function identical to equation 7. However, the primary objective of their study was to determine *E*_*max *_for relative quantification, based on nonlinear regression analysis. This contrasts the kinetic approach taken in this study, in which absolute quantification is based on converting fluorescence readings into target quantity using equation 6.

### Implementation of LRE quantification

#### Initial setup

With equations 6 and 7 in hand, the practical and analytical capabilities of LRE quantification can be tested. Furthermore, the computational simplicity of the methodology makes it amenable to manual implementation using a spread sheet. Additional data file 2 contains the MS Excel templates used for the data analysis conducted in this study. The general approach involves a series of steps that culminates in a recursive process in which the conformity of individual fluorescence readings is used to optimize the analysis.

Fluorescence readings are first imported into the spreadsheet and background fluorescence subtracted, as estimated by averaging 6–12 baseline cycles (i.e. before amplicon DNA becomes detectable). Replicate F_C _datasets are then averaged (see below), cycle efficiency (E_C_) calculated for each cycle using equation 5, and E_C _plotted against F_C _to generate what is called the "LRE plot" (Figure 2B, D and 2F). A contiguous group of points is then selected for linear regression analysis, from which estimates of *ΔE *(slope) and *E*_*max *_(intercept) are obtained, a process called "LRE analysis". Referred to as the "LRE window", this region is elemental to defining the sigmoidal model of an amplification profile. The linear integrity of the LRE window is thus crucial to the quantitative accuracy of the assay.

Various methods for selecting the LRE window size and position have been tested. In practice, details of LRE window selection for many amplicons are somewhat inconsequential, due to the high degree of conformity that can be generated. Nevertheless, as noted earlier, later cycles of some amplification profiles do not conform well to sigmoidal modeling. This is apparent in Figure 2B, D and 2F in which points corresponding to cycles within the upper region of each amplification profile "drift" off the LRE line. A key objective is therefore to avoid inclusion of these non-conforming cycles into the LRE window, while at the same time selecting the largest possible LRE window in order to maximize the precision of the linear regression analysis.

A logical starting point is to initially select a small LRE window positioned within the lower region of an amplification profile, and to progressively add consecutive cycles to the LRE window (i.e. to expand the upper limit of the LRE window), repeating the linear regression analysis as each new cycle is added to the LRE window. This is continued until encountering a cycle that clearly does not conform, based upon divergence from the LRE line. Although this approach can be reasonably effective, a more objective method that has proven to be both simpler and more sensitive becomes evident during assessment of LRE quantitative precision. However, before this can be described, another foundational principle of LRE quantification must first be implemented.

#### Target quantification, recursive analysis and the precision of LRE modeling

**Figure 3:**
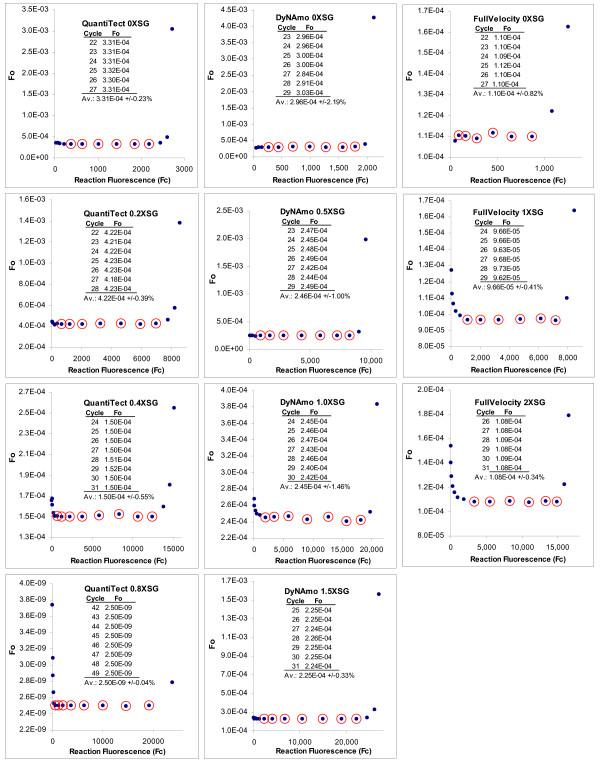
**F_0 _plots for the amplification profiles presented in Figure 2**. Following LRE analysis, individual fluorescence readings were converted into target quantity using equation 6 and the resulting values plotted back against the fluorescence readings. Numerical summaries of the F_0 _values encompassed by the LRE window (designated by red circles) are provided as inlays. The high level of similarity of F_0 _values within the LRE window is reflected by their respective CV values that range from +/-0.04% to +/-2.19%, illustrating the remarkable level of precision that can be achieved with LRE-based sigmoidal modeling. These plots also illustrate the rapid divergence in the F_0 _value generated by cycles within the upper region of these amplification profiles, due to loss of conformity with the LRE model.

A fundamental attribute of LRE quantification, as exemplified by equation 6, is the ability to convert individual F_C _readings into a target quantity, once estimates for *ΔE *and *E*_*max *_have been obtained. Indeed, several intriguing behaviors become evident when this is applied across an entire amplification profile, generating what is referred to as the "F_0 _plot". The most notable is that F_0 _values encompassed by the LRE window are very similar, a trend that can extend to F_C _readings close to the baseline fluorescence (Figure 3). In addition to providing multiple estimates of target quantity from each amplification profile, this illustrates the extraordinary precision that LRE modeling can achieve. Typically, high-quality fluorescence datasets generate a coefficient of variance (CV) for F_0 _of < ± 1.0% over a 4–6 cycle LRE window (Figure 3).

A second attribute of F_0 _plots is characterized by a rapid divergence of F_0 _values derived from later cycles within many amplification profiles. As would be anticipated, this coincides precisely with the loss in conformity to the LRE line generated in the corresponding LRE plot. Although the examples presented in Figure 3 produced a sweeping upward arc, downward arcing have also been commonly observed. In either case, the abruptness of this divergence provides a dependable marker for loss of conformity, which in turn allows the upper limit of the LRE window to be objectively defined.

Based upon the general principle of conformity, the utility of using recursive analysis to optimize the LRE window size was explored, primarily to develop an algorithm for automating LRE data analysis. The approach starts by defining a small LRE window in the lower region of a profile. Although software implementation can provide several methods for determining the lower boundary of the LRE window, manual implementation was based upon empirical determination of the lowest F_C _reading that generates sufficient precision to produce a reliable E_C _value for the first cycle of the LRE window (referred to as the LRE window "start cycle"). Linear regression analysis is conducted on this preliminary LRE window, and the F_C _readings across the entire profile converted to F_0 _(equation 6). An average F_0 _value is then calculated from the F_C _readings encompassed by the LRE window. It should be noted that this includes the F_C _generated in the cycle immediately preceding the start cycle, due to the fact that this F_C _reading is the denominator used to calculate E_C _for the start cycle.

**Figure 4:**
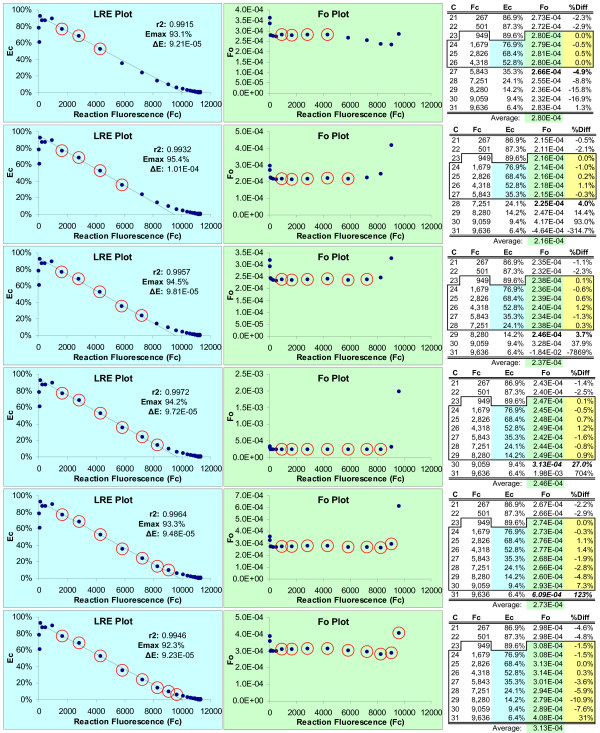
**Optimizing LRE window size via recursion**. This data is taken from the 0.5X SYBR Green I DyNAmo amplification profile (Figure 2); however, all the amplification profiles in Figure 2 generate similar results. The recursive analysis starts by assigning a small LRE window (designated by red circles) in the lower region of the amplification profile and conducting linear regression analysis using the points within this preliminary LRE window (blue highlight). F_C _readings are then converted to F_0 _using equation 6 and the average determined for F_0 _values encompassed by the LRE window (green highlight). Conformity of the F_0 _value generated by the cycle immediate following the last cycle of the LRE window is then assessed (bold), based upon the percent difference with the LRE window F_0 _average (%Diff, yellow highlight). If this difference is less than a specified threshold, the LRE window is expanded to include the next cycle and the analysis repeated. This recursive process is continued until a nonconforming cycle is encountered as defined by the threshold. Note that for illustrative purposes, the LRE window in this figure has been expanded beyond the threshold used in this study (7.5%). Although the LRE line generated within the LRE plot can be used to assess conformity, examining the resulting F_0 _values provides a more sensitive and objective methodology, as is visually illustrated by the F_0 _plots.

The recursive nature of this approach derives from comparing the average F_0 _produced by the LRE window, to that generated by the cycle immediately following the last cycle of the LRE window. The difference, expressed as a percentage of the average F_0_, is then used to assess the level of conformity. A threshold value (7.5% difference in this study) is then used to determine whether this cycle should be subsumed into the LRE window. If so, the LRE window is expanded to include this next cycle, and the LRE analysis is repeated. This recursive process is continued until a cycle is encountered that produces a F_0 _difference larger than the threshold. An example of this recursive approach is presented in Figure 4, which provides a more detailed illustration of the process. Worthy of note are the relatively small differences (typically < 0.5 fold) in the average F_0 _produced by expanding the LRE window to include cycles that are clearly nonconforming, as is illustrated in Figure 4. Even though a more formal documentation of the robustness of LRE analysis will not be attempted here, the prototypic Java program introduced at the end of the study provides the ability to quickly assess the impact of LRE window size and position.

**Figure 5:**
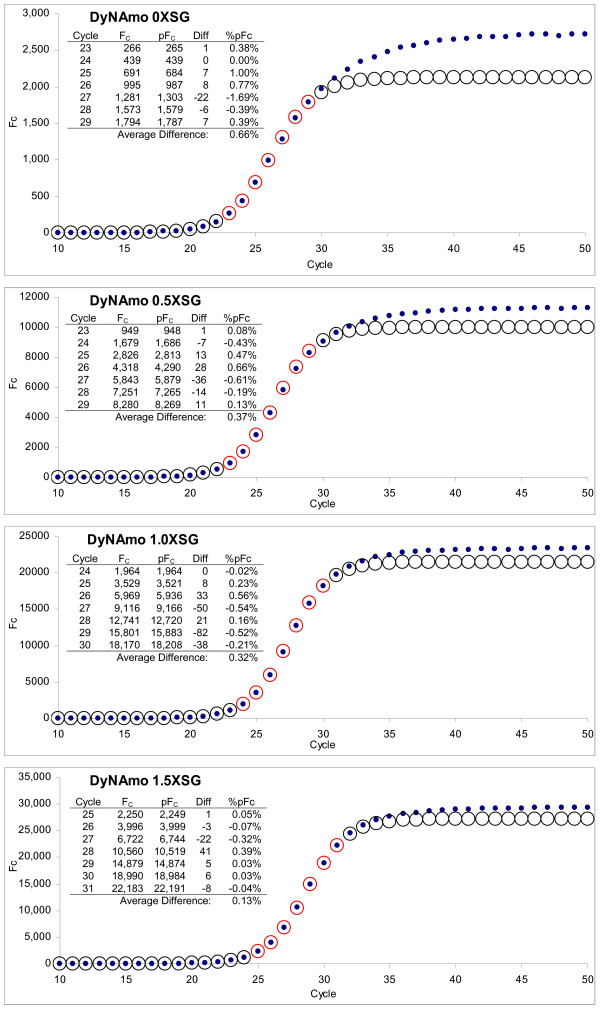
**Comparison of actual reaction fluorescence readings to those predicted by LRE modeling**. Although the DyNAmo series was selected for presentation, all three series presented in Figure 2 produced similar results. The average F_0 _calculated from the LRE window (designated by red circles, see Figure 3) was used to calculate the predicted F_C _across the entire amplification profile using equation 7 (circles) and plotted with the actual F_C _readings (dots). The numerical summaries presented as inlays illustrate the high degree of precision that can be achieved, with the average percent difference of < 0.5% for F_C _readings within the LRE window. **F**_C_: actual reaction fluorescence, **pF**_C_: predicted reaction fluorescence, **Diff**: difference between actual and predicted F_C_, **%pF**_C_: difference expressed as a percentage of the predicted F_C_.

F_0 _plots thus illustrate the two key attributes that underpin the quantitative capability of the LRE method. The first is the ability to objectively define the LRE window based on conformity of the derived F_0 _values. The second is estimating target quantity based upon averaging F_0 _values encompassed by the LRE window (typically 4–6 cycles). Furthermore, the CV of the LRE window F_0 _values is an effective indicator of the general quality of the fluorescence dataset (Figure 3), which is a major determinant of the general efficacy of LRE-based quantification (see below). Another indicator of the precision that can be achieved with LRE modeling is the correlation between actual F_C _readings and that predicted by equation 7. The profiles presented in Figure 5 exemplify high-quality datasets, which can generate predicted F_C _values that differ on average < 0.5% of the actual F_C _readings within the LRE window.

#### Maximizing optical precision

Notwithstanding the high degree of precision that can be achieved, it became evident during the early stages of LRE implementation that a number of optical factors can compromise, sometimes severely, the quality of a fluorescence dataset. Before addressing further the quantitative capability of LRE analysis, the role of reaction fluorescence determination and assay optics should first be considered. As might be anticipated, optical precision is a central determinant of the quantitative accuracy and reliability that can be achieved with LRE quantification. Two simple steps can be taken to increase optical precision. The first is to take multiple fluorescence readings at the end of each cycle (three in this study) and to use the average for determining reaction fluorescence. This reduces the error-of-measurement produced by the instrument's optical system. The second is to conduct technical replicates for each sample (four replicates in this study) and to construct a single F_C _dataset by averaging the fluorescence readings generated by the replicates.

Although conducting LRE analysis on each individual replicate amplification profile and averaging the resulting ΔE, E_max _and F_0 _values generally produces comparable results, averaging F_C _readings from replicate reactions prior to LRE analysis can increase F_C _precision substantially, particularly for F_C _datasets of marginal quality. An exception is for samples containing < 10 target molecules, due to the fact that Poisson distribution generates extensive scattering of the replicate amplification profiles, such that F_C _averaging becomes less effective.

Another key aspect is to monitor run-to-run optical variances by including a quantitative standard within each run. As described in the next section, lambda gDNA has proven to be a reliable quantitative standard that allows monitoring of the many factors impacting assay optics, through the ability to express the fluorescence intensity of an assay in quantitative terms. This introduces the concept of calibrating the optical component of real-time qPCR, which is the last foundational principle of LRE quantification.

#### Derivation of absolute scale

One of the principal attributes of employing a universal quantitative standard in combination with SYBR Green I detection is the ability to calibrate fluorescence intensity by expressing assay fluorescence in terms of amplicon mass. Referred to as "optical calibration", this can be accomplished by amplifying a known quantity of a standard and dividing the resulting F_0 _value by the predicted target quantity, expressed as nanograms of amplicon DNA (M_0_). For lambda gDNA, this takes the form:

(8) $$M_{0}=\frac{ngLambda\times A_{S}}{48,502}$$

where *ngLambda *is the mass of lambda gDNA in nanograms, *A*_*S *_is the amplicon size and 48,502 is the genome size of lambda (both expressed in base pairs) so that:

(9) $$OCF=\frac{F_{0}}{M_{0}}$$

where *OCF *is defined as the "optical calibration factor", expressed in this study as fluorescence units per nanogram of double-stranded DNA (FU/ng dsDNA). The ability to express fluorescence intensity in quantitative terms provides a simple, centralized quality control component that incorporates all of the many factors impacting the optics of an assay. These include factors related to reaction setup, such as batch-to-batch variations in enzyme formulation, reaction vessels and closures, in addition to the performance of the optical system.

**Figure 6:**
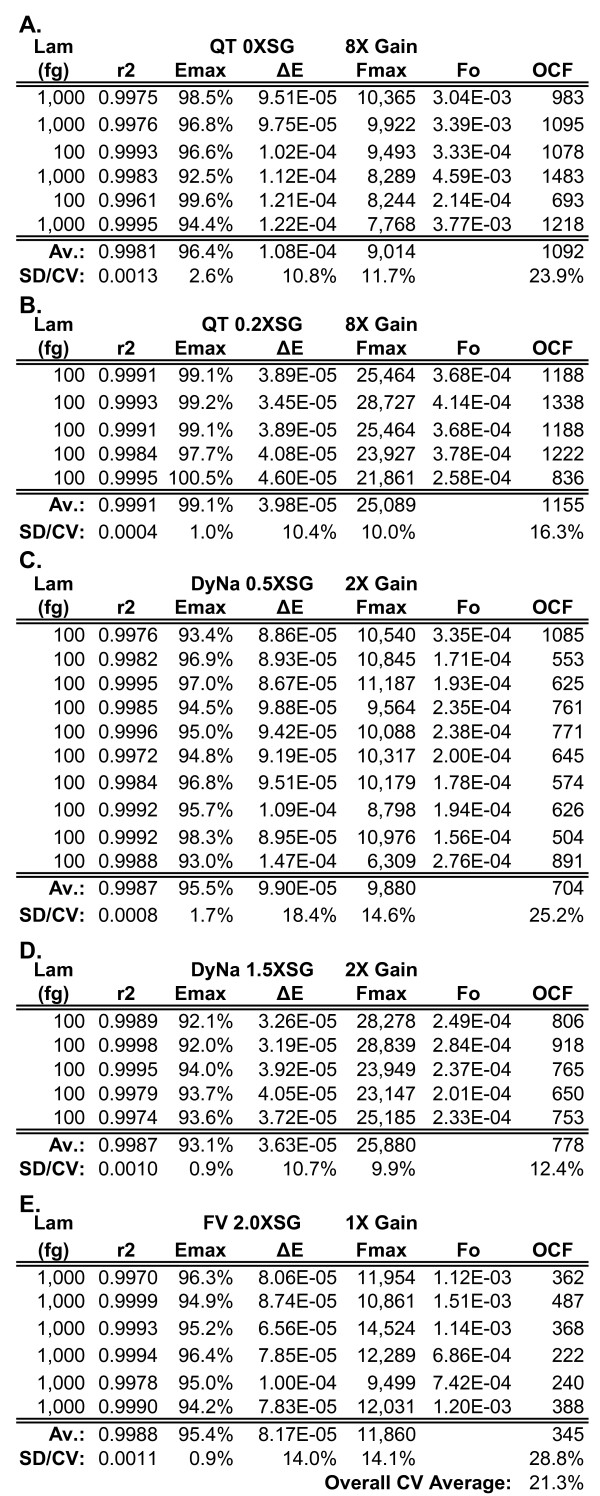
**Optical calibrations of five reaction formulations supplemented with various quantities of SYBR Green I**. Spreadsheet summary of the lambda gDNA (primer pair K7B-K12) optical calibrations conducted for the five reaction formulations used for LRE quantification in this study. Based on data presented in Figure 2, these formulations were designed to assess the impact of SYBR Green I quantity, the highest supplemented quantity (2.0X) estimated to be > 10X than that present in these three commercial formulations. Each row of values was derived from an individual amplification run (see additional file 3 for more details). **(A) **QuantiTect 0X SYBR Green I. **(B) **QuantiTect 0.2X SYBR Green I. **(C) **DyNAmo 0.5X SYBR Green I. **(D) **DyNAmo 1.5X SYBR Green I. **(E) **FullVelocity 2.0X SYBR Green I. **Lam**: lambda gDNA quantity in femtograms, **QT**: QuantiTect, **DyNa**: DyNAmo; **FV**: FullVelocity, **SG**: SYBR Green I, **SD**: standard deviation, **CV**: coefficient of variation (SD/Average × 100%).

An illustration of this approach is presented in Figure 6, which summarizes optical calibrations conducted with five reaction formulations supplemented with various quantities of SYBR Green I. Derived from the analyses of 32 individual amplification runs, this large dataset provides a general indication of the variances generated by each LRE parameter, culminating in an OCF average CV of ± 21.3% across all five reaction formulations. All things being equal, this should be indicative of the resolution that a LRE-based quantitative assay can achieve. Indeed, similar variances were generated by repeated quantifications of eleven mRNA targets, as described in the next section. Of greater significance, however, is the ability to use optical calibration to establish an absolute quantitative scale.

The concept of optical calibration was first introduced within the context of standard curve-based quantification, in which it was recognized that correlating reaction fluorescence to DNA mass could provide a simplified method for establishing quantitative scale [9]. Optical calibration in a form similar to that presented here was subsequently described within the context of absolute quantification using nonlinear regression (SCF) [18]. In either case, it was evident that the ease of conducting absolute qPCR would be greatly increased by implementing optical calibration using a universal quantitative standard. In addition to eliminating the need to prepare target-specific standards, when combined with LRE analysis optical calibration circumvents the need to construct standard curves.

Under this approach, absolute quantification is achieved by first converting the derived F_0 _value into DNA mass using a lambda-based optical calibration factor (equations 8 and 9):

(10) $$M_{0}=\frac{F_{0}}{OCF}$$

for double-stranded DNA targets, or:

(11) $$M_{0}=\frac{F_{0}}{\left(OCF\times 0.5\right)}$$

for single-stranded DNA targets, where *M*_0 _is target mass expressed as nanograms of amplicon DNA. Conversion into the number of target molecules then simply requires relating M_0 _to the molecular size of the amplicon:

(12) $$N_{0}=\frac{M_{0}\times 9.1\times 10^{11}}{A_{S}}$$

were *N*_0 _is the number of target molecules, *A*_*S *_is amplicon size in base pairs, and 9.1 × 10^11 ^is the number of base pairs per nanogram of dsDNA [18]. In view of the operational simplicity provided by this approach, few additional requirements would be necessary to fully automate absolute quantification. However, one important caveat remains to be addressed, which is the implicit assumption that all amplicons generate similar fluorescent intensities.

### Application to gene expression profiling

Key to utilizing a single absolute scale across multiple targets is the underlying assumption that amplicon-specific factors such as amplicon size and/or base pair composition, do not significantly impact the intensity of SYBR Green I fluorescence (i.e. FU/bp). One approach to testing this assumption would be to compare quantifications generated by a large number of diverse amplicons targeted to a quantified standard (such as lambda gDNA), with the expectation that differences in optical intensity would generate quantitative biases. Although such an analytic approach can be effective, the approach chosen for this study was based upon applying LRE quantification to gene expression analysis. An in-house initiative to develop large-scale expression profiling in *Arabidopsis thaliana *provided a large database from which to select candidate targets for absolute quantification. Eleven transcripts were selected based primarily on encompassing a quantitative range indicative of transcriptional factors, which for this group of targets was estimated to be 10–10,000 transcript molecules per 10 ng of total RNA. Furthermore, these targets encompass amplicon sizes of 85–150 bp and amplicon GC contents ranging from 40–53% (see Methods for further details), from which it was expected that any substantive differences in amplicon fluorescence would generate recognizable quantitative biases.

**Figure 7:**
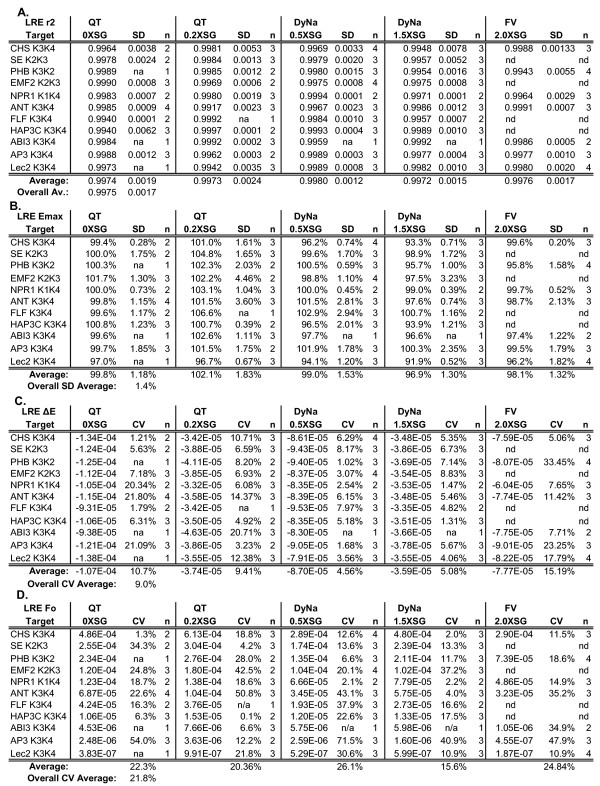
**LRE analysis of eleven cDNA targets amplified with five reaction formulations**. Summaries for LRE analysis of a total of 134 amplification profiles (each profile an average of four replicate amplification reactions) generated from eleven cDNA targets quantified using five reaction formulations (see Figure 2 and additional file 3 for additional details). **(A) **Average linear regression correlation coefficients (r^2^). **(B) **Average E_max_. **(C) **Average ΔE. Note that ΔE is dependent on the optics of the assay and are thus not directly comparable across different formulations and gain settings. **(D) **Average F_0 _derived from the average of F_0 _values encompassed by the LRE window (see Figure 4 for details). **QT**: QuantiTect, **DyNa**: DyNAmo; **FV**: FullVelocity, **SG**: SYBR Green I, **SD**: standard deviation, **n**: the number of runs, **na**: not applicable (only one run conducted), **nd**: not determined.

The most notable initial outcome was that all amplicons generated amplification profiles that conformed well to the LRE model, as reflected by an average LRE linear regression correlation coefficient (r^2^) of > 0.995 (Figure 7A, additional file 3). Indeed, of the > 400 primer pairs tested to date, all have conformed well to LRE modeling. Unexpectedly, increasing SYBR Green I quantity had little or no impact on the r^2 ^of the LRE analysis, despite a greater than X10 increase in F_max _at the highest SYBR Green I quantity examined (Figure 7A). Increasing SYBR Green I quantity also did not reduce the quantitative variance of the derived F_0 _values (see below).

This high level of precision is further reflected by the low variances generated by the corresponding *E*_*max *_and *ΔE *determinations (Figure 7B and 7C). Worthy of note is the standard deviation of *E*_*max *_determination, which averaged ± 1.4% across all targets and reaction formulations. In addition to demonstrating the robust nature of LRE analysis, such low variances support the contention that PCR amplification is an inherently precise process. Furthermore, without delving into the mathematics of the LRE model, it should be noted that E_max _has by far the greatest impact on target quantification (a 1% difference in E_max _roughly produces a 20–30% difference in the calculated target quantity), such that *E*_*max *_estimation is the primary determinant of the quantitative resolution that can be achieved. Based upon the low variances in *E*_*max *_and *ΔE *determinations, it is not surprising that the derived F_0 _values for each target generated an overall average CV of ± 21.2% (Figure 7D). As noted earlier, this is very similar to the average OCF CV of ± 21.3%, indicating that LRE has the potential to resolve quantitative differences of < ± 25%. Quantitative resolution also appears not to be impacted by increasing SYBR Green I quantity, with each of the five reaction formulations producing similar F_0 _CVs (Figure 7D).

This dataset also illustrates the limitations of expressing target quantities in units dependent on assay setup and instrument optics. In this case, it is not possible to directly compare F_0 _values across different enzyme formulations, due to differences in reaction intensity and gain settings. However, converting F_0 _quantities into the number of target molecules provides an absolute context that allows comparison of target quantities generated under disparate assay conditions. Although this may seem obvious, it is worth stressing that provision of a universal context is what makes absolute quantification so compelling, in that absolute values transcend issues of assay design, instrumentation, and even the type of data analysis applied. Table 1 provides an illustrative example, which demonstrates that SYBR Green I quantity and enzyme type had a modest, if any, impact on LRE quantification. Furthermore, the average CV of ± 23.4% produced across all five reaction formulations is in general agreement with the contention that LRE quantification is able to resolve quantitative differences smaller than ± 25%.

**Table 1:** LRE-based absolute quantification of eleven cDNA targets using five enzyme formulations.

Gain:	X8	X8	X2	X2	X1		
OCF:	1092	1155	704	778	345		
Enzyme:	QT 0XSG	QT 0.2XSG	DyNa 0.5XSG	DyNa 1.5XSG	FV 2.0XSG	LRE-No Average	CV (+/-)
Target							
CHS K3K4	6,143	7,323	5,658	8,500	11,598	7,844	30.2%
SE K3K2	4,828	5,444	5,103	6,363	nd	5,435	12.3%
PHB K3K2	2,951	3,292	2,646	3,733	2,956	3,116	13.3%
EMF2 K2K3	2,301	3,256	3,094	2,752	nd	2,851	14.8%
NPR1 K1K4	1,872	1,973	1,565	1,657	2,334	1,880	16.0%
ANT K3K4	970	1,390	756	1,139	1,446	1,140	25.3%
FLF K3K4	661	553	466	596	nd	569	14.4%
HAP3C K3K4	145	198	254	255	nd	213	24.7%
ABI3 K3K4	66	105	129	122	48	94	37.8%
AP3 K3K4	38	52	61	34	22	41	37.3%
Lec2 K3K4	6	14	12	12	9	11	31.4%

						Av. CV:	23.4%

Following LRE analysis (summarized in Figure 7), the F_C _readings encompassed by the LRE window were converted to F_0 _(see Figure 3) and the average F_0 _value converted to the number of target molecules (LRE-N_0_) using the OCF generated by each respective reaction formulation (see Figure 6 and additional file 3 for more details). The gain setting was adjusted to prevent saturation of the photomultiplier (see Figure 2). Gain: the photomultiplier gain setting, OCF: optical calibration factor (FU/ng dsDNA), QT: QuantiTect, DyNa: DyNAmo; FV: FullVelocity, SG: SYBR Green I, SD: standard deviation, nd: not determined.

Notwithstanding the high level of precision that can be achieved, it is important to note that despite expressing quantities as the number of target molecules, the quantitative context is still confined to the LRE analyses used to generate the dataset. That is, this type of comparison is unable to verify absolute accuracy, in that any biases generated by LRE analysis and/or optical calibration will generate quantitative biases. As is presented in the following two sections, the quantitative context can be expanded by determining how closely target quantities produced by other methods correlate with the LRE-derived quantities.

### Absolute quantification via C_t_

Positional analysis, as exemplified by the threshold method, has predominated since the introduction of real-time PCR over 15 years ago [35], and is the quantitative methodology upon which all commercial platforms currently rely. Based upon the fractional cycle at which reaction fluorescence reaches a threshold value, the threshold method defines profile position through a common reference point called the threshold cycle or "C_t_". Absolute quantification is accomplished via standard curves constructed with target-specific quantified standards [9], which are technically challenging and prone to generating quantitative errors. However, previous recognition that a sigmoidal-derived E_max _is analogous to a slope-derived amplification efficiency generated from a standard curve [18] suggests an alternative method for converting C_t _values into target molecules, using the exponential equation:

(13) $$F_{0}=\frac{F_{t}}{{\left(E_{\max}+1\right)}^{C_{t}}}$$

were *F*_*t *_is the fluorescence threshold used to derive *C*_*t*_, and *E*_*max *_is the amplification efficiency derived from LRE analysis. Importantly, this approach allows the application of optical calibration so that C_*t*_-based absolute quantification can be conducted without standard curves.

Table 2 summarizes the target quantities produced by this approach using the C_t _values generated from the amplifications summarized in Figure 7 (see additional file 3 for more details). Similar levels of quantitative variance were seen across all five reaction formulations as compared with those produced by LRE quantification (Table 1). Not only does this confirm the high level of quantitative precision that can be achieved with real-time qPCR, it provides the opportunity to further extend the quantitative context by comparing C_*t*_- and LRE-based quantifications.

**Table 2:** Absolute quantification based upon conversion of C_t _to F_0 _via E _max _and F_t_.

Gain:	X8	X8	X2	X2	X1		
OCF:	1092	1155	704	778	345		
Enzyme:	QT 0XSG	QT 0.2XSG	DyNa 0.5XSG	DyNa 1.5XSG	FV 2.0XSG	LRE-No Average	CV (+/-)
Target							
CHS K3K4	5,876	7,562	5,564	9,188	10,813	7,801	28.5%
SE K2K3	4,874	5,919	5,255	7,485	nd	5,883	19.6%
PHB K3K2	2,653	3,405	2,730	3,916	2,888	3,119	17.1%
EMF2 K2K3	2,256	3,271	2,960	2,821	nd	2,827	15.0%
NPR1 K1K4	2,039	2,070	1,634	1,892	2,369	2,001	13.4%
ANT K3K4	923	1,288	972	1,342	1,450	1,195	19.6%
FLF K3K4	442	651	489	665	nd	562	20.1%
HAP3C K3K4	148	195	255	285	nd	221	27.7%
ABI3 K3K4	65	108	139	139	46	100	42.6%
AP3 K3K4	32	54	40	39	26	38	27.6%
Lec2 K3K4	3	14	11	11	8	10	41.7%
						Av. CV:	24.8%

C_t _values generated by the amplification profiles summarized in Figure 7 were converted to F_0 _using equation 6, and then converted to target molecule number via the OCF as used for LRE quantification (see text and additional data file 3 for additional details).

**Figure 8:**
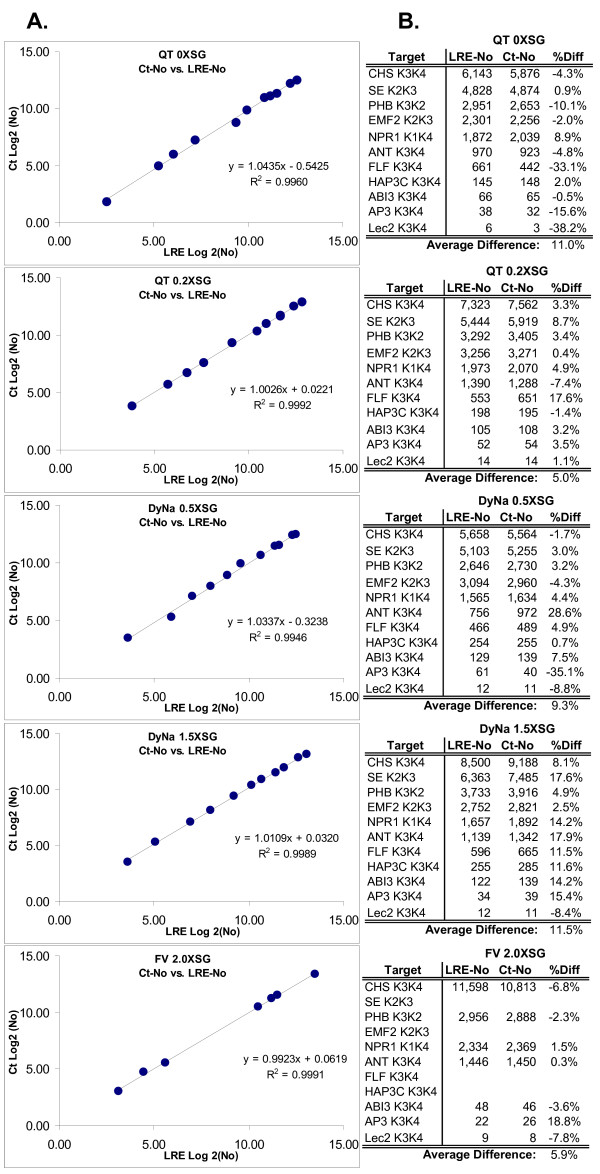
**Comparison of LRE- and C_t_-based quantifications**. **(A) **Log_2 _plot of C_t _vs. LRE quantifications using five reaction formulations summarized in Figure 7. **(B) **Numerical summary. **R2**: linear correlation coefficient, **%Diff**: difference expressed as a percentage of LRE-No.

Figure 8 presents Log_2 _plots and numerical summaries comparing LRE and C_t _derived quantities of the eleven cDNA targets, for each of the five reaction formulations. The high level of correlation reflected by an average r^2 ^of > 0.995 provides strong corroborating evidence for the precision of LRE quantification. An important qualification, however, is that C_t _quantification conducted in this fashion is dependent upon both E_max _and OCF. As such, any quantitative biases generated by LRE analysis will impact the absolute accuracy of both LRE- and C_t_-based quantifications. It should be noted that standard curves are also susceptible to introducing quantitative biases, produced for example, by errors in quantification of the standard and/or preparation of the dilution series. Importantly, this highlights the dilemma associated with any real-time qPCR assay, irrespective of how it is implemented; that is, how to effectively assess the true quantitative accuracy, referred to in this study as "absolute accuracy".

Accuracy is typically determined by repeatedly measuring some traceable reference standard. While this approach could be effective for well-characterized targets, the use of reference standards is impractical for applications involving large numbers of diverse targets. What would be more effective is an alternative quantitative method that would, as much as possible, be free from potential biases generated by real-time qPCR. A superbly effective solution has been provided by Wang and Spadoro [36], which exploits the single-molecule sensitivity inherent to PCR amplification. Founded upon the principles of Poisson distribution, this approach provides an elegant means for absolute quantification that would be familiar to any microbiologist or virologist; that is, the method of "limiting dilution assay".

### Absolute quantification via limiting dilution assay (LDA)

The efficacy of LDA derives from its ability to achieve absolute quantification independent of the kinetic and optical principles upon which real-time qPCR is dependent, and does not require a quantitative standard. LDA relies solely on the frequency of reactions that fail to produce an amplification product, utilizing PCR to only determine whether a target molecule is present within an individual aliquot. Furthermore, the assay is not dependent on how the PCR amplification is actually conducted, requiring only that it generate single-molecule sensitivity, and that amplification reactions that generate false positives (e.g. primer dimers) are either absent or can be identified. Thus, an economical SYBR Green I-based LDA could, for example, be used to evaluate the quantitative accuracy of a probe-based assay.

As the name implies, LDA involves diluting the sample to a limit, which in this case are individual target molecules. As dictated by Poisson distribution, when a sample is diluted to a point near to one target molecule per aliquot, a high proportion of aliquots will not contain a target molecule. It is the frequency of these "nil" aliquots that allow the average target quantity to be calculated using the equation:

(14) Average target molecules per aliquot = -Ln(Nil/Total)

where Nil and Total are the number of reactions that fail to produce amplicon DNA and the total number of reactions conducted, respectively [36]. Target quantity is then calculated based on the dilution factor used to reach single a molecule concentration. It is important to note that LDA is self-validating, such that if the sample is under-diluted no nil reactions will be produced, whereas if the sample is over-diluted all reactions will be nil. As a result of this intrinsic self-validation, LDA has in practice proven to be exceptionally reliable.

**Figure 9:**
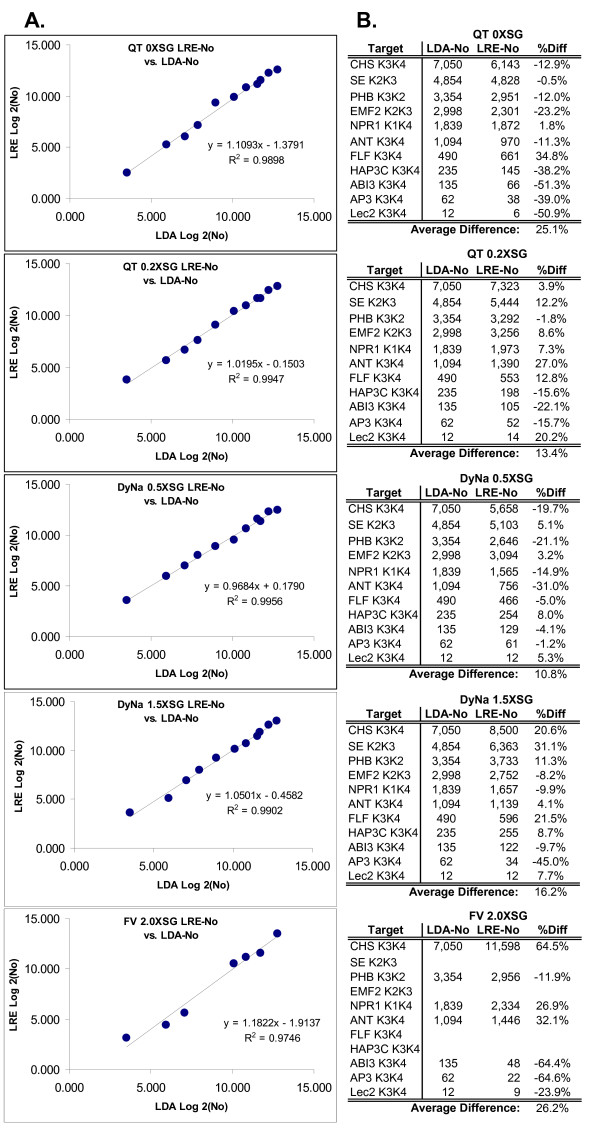
**Assessment of quantitative accuracy via Limiting Dilution Assay (LDA)**. **(A) **Log_2 _plot of LRE vs. LDA quantifications. **(B) **Numerical summary. **R2**: linear correlation coefficient, **%Diff**: difference expressed as a percentage of LDA-No.

Table 3 summarizes results from LDAs conducted on all eleven mRNA targets, for which the dilution factor applied was based upon the LRE quantification for each respective target. Comparing the LDA quantifications to the LRE quantifications via Log_2 _plots, four of the five reaction formulations generated an r^2 ^> 0.99, with the FullVelocity formulation generating an r^2 ^of 0.97 (Figure 9). Excluding the FullVelocity dataset, this translates to an absolute difference of < ± 20% on average. Overall, this dataset not only illustrates the level of absolute accuracies that can be achieved with LRE quantification, but also indicates that any quantitative biases generated by differences in amplicon fluorescence intensity are most likely to be small.

**Table 3:** Absolute quantification via limiting dilution assay.

	Dilution	Total	Nil	N per	
Target	Factor	Rxns	Rxns	Aliquot	LDA-No
CHS	15,000	48	30	0.47	7,050
SE	6,222	48	22	0.78	4,854
PHB	6,222	48	28	0.54	3,354
EMF2	8,000	48	33	0.37	2,998
NPR1	3,000	48	26	0.61	1,839
ANT	1,250	48	20	0.88	1,094
FLF	500	24	9	0.98	490
HAP3C	466	48	29	0.50	235
ABI3	250	48	28	0.54	135
AP3	67	48	19	0.93	62
Lec2	20	48	27	0.58	12

Based upon the N_0 _values produced by LRE quantification, a sample of each reverse transcriptase reaction was diluted to a predicted 0.5–1.0 target molecules per aliquot for each of the eleven cDNA targets, and replicate amplification reactions were conducted to determine the frequency of aliquots lacking a target molecule (Nil Rxns). The number of target molecules (N) per aliquot was then calculated using equation 14 and the predicted number of target molecules in the reverse transcriptase reaction (LDA-No) was calculated by multiplying by the dilution factor (see text for additional details).

### Recognized anomalies impacting LRE quantification

During the development and testing of LRE quantification, a number of factors were identified that either compromise or prohibit effective application of LRE quantification. Referred to here as "anomalies", these actually encompass a broad variety of factors, ranging from primer pair performance to optical precision and data processing of fluorescence readings.

#### Compromised efficiency of target priming and elongation

A fundamental principle impacting the quantitative accuracy of real-time qPCR is simple in principle, but not necessarily apparent; that is, disrupting target priming and elongation. Best exemplified by single nucleotide polymorphisms (SNPs), base-pair mismatches between a primer and the target can severely disrupt initiation of PCR amplification, but once an amplicon molecule is formed these mismatches are lost, such that amplicon amplification proceeds unhindered. LRE analysis of the resulting amplification profile is thus incapable of detecting target priming anomalies. Indeed, any factor that disrupts the efficiency of target priming and elongation that does not equally reduce the efficiency of amplicon priming and elongation, will necessarily generate an under-estimation of target quantity.

Such a situation can be prevalent for species or genotypes where SNP occurrence is undocumented. A simple but effective solution is to compare quantifications generated by multiple amplicons per target (see Methods for additional details). Disruption of target priming and elongation then becomes evident as a shift to later cycles, of the profile generated by the impacted primer(s). Although not yet formally documented, preliminary work has also indicated that certain PCR inhibitors can also selectively disrupt target priming and elongation, an issue that is fundamentally more difficult to address.

#### Compromised optical integrity

Factors impacting assay optics can severely compromise LRE quantification. A simple example encountered during an early set of experiments, was a problem eventually traced back to optical variance in the reaction tubes, which produced up to a 40% difference in fluorescence intensity between identical amplification reactions (data not shown). As described earlier, monitoring assay optics via amplification of quantitative standard such as lambda gDNA, has proven effective for identifying such optical anomalies via differences in the resulting OCF values.

The initial processing of fluorescence datasets is another factor that can impact optical precision. It is paramount to ensure that beyond fluorescence background subtraction, no additional modifications are performed on the F_C _datasets. The most commonly encountered data manipulation is often referred to as "curve smoothing" in which a running average is applied in order to generate more aesthetically pleasing amplification profiles. Even though the apparent sigmoidal character of the resulting profiles can be improved (i.e. increased linearity of the LRE plot), such modifications significantly distort LRE analysis, the most evident being a large reduction in E_max_. Some forms of optical normalization could also be expected to be problematic, although this has not been formally investigated. As a general rule, it is recommended that as few modifications as possible be conducted on F_C _datasets, even to the point of conducting background subtraction manually in order to ensure an accurate baseline estimate, as was necessary in this study (see Methods for additional details).

#### Baseline drift

Another form of F_C _data modification attempts to correct one of the most acute anomalies we have recognized to date; that is changes in the background fluorescence. Referred to as "baseline drift", we have found that some primer pairs, in addition to sample-specific factors can produce a progressive increase in fluorescence background. Importantly, some data processing packages attempt to correct for fluorescence drift by adjusting the values of the F_C _readings. While this can be somewhat effective in reducing the quantitative inaccuracies produced by baseline drift, it also leads to unintended distortions that remain hidden if the user is unaware of the underlying F_C _manipulation (see Methods for additional details). Baseline drift can also be quite subtle and is most effectively recognized by visual examination of raw fluorescence data before subtraction of background fluorescence.

**Figure 10:**
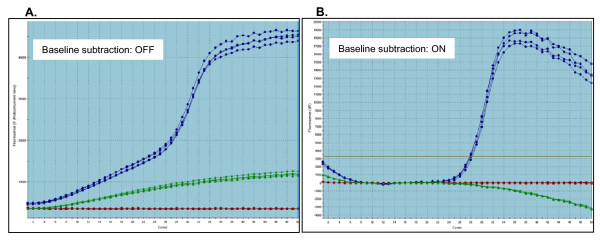
**An example of primer-specific baseline drift**. An extreme example of baseline drift produced by lambda primer K30. **(A) **Amplification profiles of 100 femtograms of lambda gDNA with K23 and K30 primers (blue profile), K30 alone with no lambda gDNA (green profile) and K23 alone with no lambda gDNA (red profile) with no baseline subtraction. **(B) **The same profiles following baseline subtraction (F_b _= average F_C _of cycles 7–18). This illustraties the distortions that can be produced by the MxPro software used in this study, which attempts to correct for the baseline drift by modifying the F_C _dataset. This can compromise, sometimes severely, the efficacy of LRE quantification, even in the absence of any apparent baseline drifting (data not shown)

Our investigation into the source of baseline drift using SYBR Green I detection has revealed it to be a complex phenomenon, likely generated by several mechanisms. One clear trend is a strong primer-specific effect (Figure 10). Unfortunately, repeated attempts to identify reliable predictors, such as sequence complementarity or secondary structural elements (e.g. hairpin loops) within the primers, have to date been largely unsuccessful. Unidentified sample-specific factors have also been found to generate extensive baseline drift across multiple primer pairs that normally do not generate baseline drift. Raising the annealing and elongation temperature and/or diluting the sample, as well as re-purifying the RNA before conducting reverse transcription, have been found to reduce baseline drifting in some cases.

### Automation

**Figure 11:**
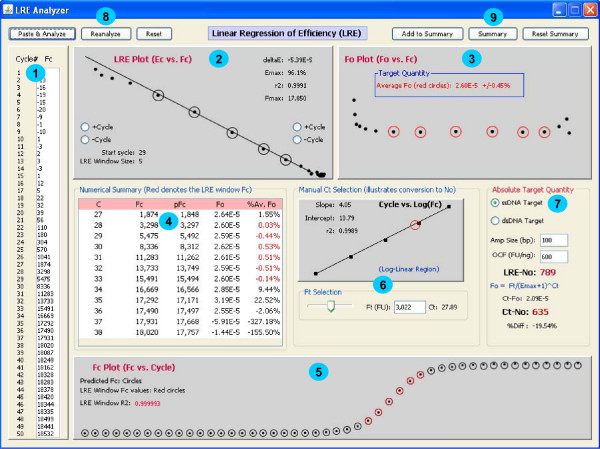
**Screen shot of a prototypic Java program that automates LRE quantification**. Made up of several panels that bring together all of the components of LRE quantification, this program allows the interrelationships of each component and their impact on the calculated target quantity to be examined. **1**. Lists the F_C _dataset. **2**. The LRE plot which allows manual adjustment to the LRE window (denoted by circles). **3**. The F_0 _plot from which target quantity is derived by averaging the F_0 _values within the LRE window (denoted by red circles). **4**. Numerical summary with the red lettering denoting the F_C _readings encompassed by the LRE window. **5**. The F_C _plot which compares actual (dots) to predicted (circles) F_C_, with the red circles denoting the F_C _readings encompassed by the LRE window. **6**. C_t _determination based upon user-selected fluorescence threshold based on linear regression analysis of the log-linear region of the amplification profile (implementation details available from the corresponding author upon request). **7**. Target quantification where the number of target molecules is calculated based upon four parameters: average F_0 _taken from 3, whether the target is double or single stranded, the amplicon size, and the optical calibration factor (OCF) (see text for details). Conversion of C_t _to N_0 _via F_0 _is also included, which allows the quantitative differences between LRE- and C_t_-based quantification to be assessed. **8**. Buttons provide the ability to paste a F_C _dataset from the clipboard and to initiate automated analysis ("Paste & Analyze"), or to repeat the automated analysis on the current F_C _dataset ("Reanalyze"), or to reset the F_C _dataset to the default provided with the program ("Reset"). **9**. Provides a summary presented as a dialog box that that can be copied to clipboard and pasted into Excel.

LRE quantification is primarily a matter of data processing. In practice, the only component that requires any significant insight is LRE window selection. If implementation of LRE analysis could be relegated to a computer program, the user would only need to supply the size of the amplicon and an optical calibration factor in order to complete the quantification. A prototypic Java program (additional file 4) supports this contention by automating start cycle selection and optimizing LRE window size using the recursive approach described earlier. This program brings together all of the elements of LRE quantification, providing the opportunity to examine the interrelationships between LRE window selection, the resulting F_0 _and N_0 _values, and the predicted F_C _values generated by the LRE model. This includes the ability to manually adjust the LRE window, to change the associated C_t _value by adjusting the fluorescence threshold, and to enter values for the OCF and amplicon size (Figure 11). Entry of an F_C _dataset via pasting from clipboard also provides limited data processing capabilities.

### Comparison to other automated data processing packages

To further examine the performance capabilities of LRE, the automated LRE quantification provided by the prototypic Java program was compared to two other publicly available automated data processing packages, which also analyze the F_C _readings generated by individual amplification reactions. The first called LinReg, has become a commonly used package for determining amplification efficiency without the use of a standard curve, and employs linear regression analysis of the log-linear region present within the lower region of an amplification profile. Based upon the presumption that amplification efficiency is constant within this region, amplification efficiency is determined from the slope of a Log(F_C_) vs. cycle plot, with target quantity (F_0_) determined by the intercept, that is, when cycle = 0 [12]. The second package called Miner [15] determines amplification efficiency via nonlinear regression using the same exponential model upon which LinReg is based. Miner also generates a C_t _value based upon a dynamic fluorescence threshold (F_t_). Although Miner does not provide an F_0 _value, equation 13 allows a F_0 _value to be calculated using the amplification efficiency, F_t _and C_t _values generated by Miner.

The approach taken for the comparison was to select a single, representative amplification profile for each of the eleven cDNA targets, across all five assay formulations. This provided a total of 55 amplification profiles for analysis, which are provided in additional file 5. Each amplification profile was then subjected to automated analysis by each of the three packages using default values, from which an amplification efficiency estimate and an F_0 _value were obtained (additional file 6). The ultimate objective, however, was to assess the absolute quantitative accuracies by comparison to the LDA quantifications presented in Table 3. In order to accomplish this, it was first necessary to conduct individual optical calibrations for each of the three analysis packages, in order to compensate for the biases specific to each package. Most notable is that both LinReg and Miner generated amplification efficiency estimates that were generally lower than that produced by LRE (additional file 6). All of the lambda gDNA amplification profiles (additional file 7) were thus subjected to analysis by each of the three packages, producing an optical calibration factor specific to each analysis package for each of the five assay formulations. F_0 _values were then converted to the number of target molecules using the corresponding OCF, as summarized in additional file 6.

**Figure 12:**
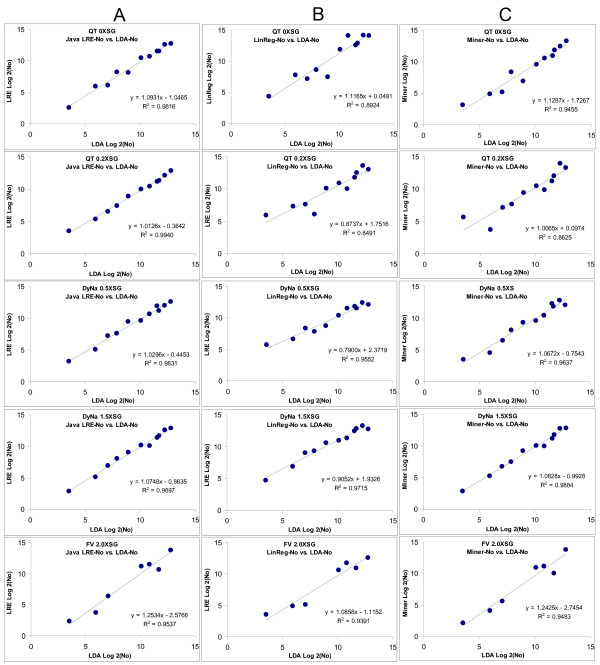
**Comparison of automated LRE quantification with LinReg and Miner via comparison to LDA quantification**. Amplification profiles for each of the five enzyme formulations representative of all eleven transcripts (additional file 5) were subjected to analysis with: **(A) **Java-LRE analysis using the prototypic Java program that automates LRE analysis (additional file 4) **(B) **LinReg which determines amplification efficiency and F_0 _via linear regression using the F_C _readings within the log-linear region. **(C) **Miner which determines amplification efficiency using non-linear regression of the log-linear region, along with C_t _values based upon a dynamic F_t_. The resulting F_0 _values converted to the number of target molecules (N_0_) via optical calibration (see text). The quantities produced by each of the three methods are compared to the absolute quantification generated by limiting dilution assay (Table 3) via Log_2 _plots. Based on the resulting r^2 ^values, LRE produced the highest quantitative accuracy for all five a formulations.

Figure 12 provides a summary of the comparison, in which the quantifications produced by each package are compared to the LDA quantification via Log_2 _plots. Overall, Java-LRE performed the best, producing an average r^2 ^value of 0.984, while Miner and LinReg produced average r^2 ^values of 0.942 and 0.922, respectively. Differences in performance become more apparent when the quantitative differences are expressed as the percentage of LDA quantity, as presented in additional file 6. Both Miner and LinReg performed less well, producing 2.4X and 6.1X greater variance, respectively, than Java-LRE (additional file 6).

## Discussion

### What should be expected from real-time quantitative PCR?

Often for historical reasons expectations for quantitative PCR can differ greatly, and can be influenced as much by personal perspective as by methodological considerations. The plethora of choices currently available for detection chemistry, enzyme formulation, cycling regime and instrumentation provide many compelling examples. It is not uncommon, for example, for widely differing protocols to be applied to seemingly identical applications, with little or no supporting evidence that any single assay design is superior. Exacerbated by a profound lack of standardization, it is not surprising that many reports caution about the limitations of real-time qPCR [28,37-39].

Evaluating real-time qPCR technologies is further complicated by the fact that performance expectations can be highly context dependent, and that the application of performance standards (if any) can vary as widely as context. Biomedical diagnostics, for example, provide many poignant scenarios in which assay performance supersedes assay design. Without verifiable accuracy and reliability, methodological details can become immaterial. Arguably, gene expression analysis often represents the other extreme where, for reasons of technical simplicity, transcript quantities are frequently expressed as relative differences. In addition to providing a very limited quantitative context, relative quantification provides little or no opportunity to assess quantitative accuracy.

Despite a broad range of performance expectations, it should be evident that absolute qPCR can enhance the efficacy of any quantitative assay, irrespective of context. Not only does absolute quantification impart a universal perspective that facilitates data interpretation, it also allows assay performance to be defined in absolute terms. Furthermore, absolute quantification allows decoupling of target quantification from assay implementation, such that quantitative data generated by disparate assay designs and/or data processing methodologies can be directly compared.

Notwithstanding the apparent utility of absolute quantification, the technical complexity and resources required by current protocols is daunting. Not only has this greatly impeded broad adoption of absolute quantification, the complexities of implementing even the most basic quantitative assay hinders access to real-time qPCR technologies, particularly for casual users. Furthermore, the necessity for constructing target-specific standard curves severely limits both the efficacy and capacity of absolute quantification.

Founded on recognition that PCR amplification is inherently sigmoidal, this study describes methodologies that provide effective, and in some cases simple solutions for conducting absolute quantification without standard curves. Utilizing a kinetic-based approach, LRE analysis can be applied to any SYBR Green I-based assay, with few qualifications other than that the F_C _datasets be of reasonable quality. LRE quantification does not rely on user-supplied standards and, if automated data processing is implemented, requires little or no training beyond that required for preparing amplification reactions. Particularly in view of the impact that absolute qPCR could have on a broad range of applications, these attributes alone provide a compelling argument for moving beyond the historical, often dogmatically held concepts that have persisted since the introduction of real-time qPCR. Key to this endeavor is to develop an effective understanding of the fundamental principles of absolute quantification, many of which transcend details of assay design and data analysis methodology.

### The two founding principles of absolute quantification

Despite the seemingly complex mix of technologies and methodologies, absolute quantification requires measurement of only two fundamental parameters – amplification kinetics and quantitative scale – regardless of detection chemistry, enzymology or instrumentation. Historically, this has been accomplished by constructing target-specific standard curves, in which amplification efficiency is derived from the slope and quantitative scale is derived from the intercept [9].

#### Amplification efficiency determination

Assessing amplification kinetics has long been recognized as a major factor impacting qPCR, due to the fact that errors in amplification efficiency determination can lead to large quantitative errors. Nevertheless, early real-time qPCR protocols simplified target quantification by assuming amplification efficiency to be identical for all amplicons and all samples [40]. Indeed, although the slope of a standard curve provides an estimate of amplification efficiency, a similar assumption must still be made; that is that the amplification efficiencies of all samples are identical, or at least similar, to that predicted by a standard curve. Thus, even if a standard curve can be effectively constructed, quantitative accuracy cannot be ensured due to the potential for sample-specific factors to reduce amplification efficiency. This can be a major concern, particularly for samples originating from sources known to contain inhibitory compounds, such as for environmental samples and for many types of biomedical samples. This deficiency alone would be expected to exclude real-time qPCR from a potentially large category of applications, where unidentified quantitative errors cannot be tolerated. LRE analysis provides a fundamental solution to this issue, through the ability to monitor amplification efficiencies within individual amplification reactions.

Notwithstanding the innate limitations of standard curve-based quantification, the implications of amplification efficiency determination extend beyond issues of quantitative accuracy, playing a predominant role in the operational component of real-time qPCR. Amplification kinetics is determined by a combination of reaction setup and cycling regime. Thus, amplification efficiency must be determined for every new amplicon, and re-determined if changes are made to reaction setup and/or cycling regime. As such, difficulties in determining amplification efficiency limit the number of targets and reaction conditions that can be tested. Under this context it becomes apparent that LRE confers operational attributes important to developing reliable, high-capacity qPCR applications, which are beyond what are possible using current technologies. For example, LRE analysis allows performance assessments to be based on amplification of bona fide samples, as opposed to the common practice of using artificial targets such as plasmids or oligos, and with a capacity limited only by the number of amplification reactions that can be run. Of a more fundamental nature is the potential to extend performance assessment beyond amplification efficiency, to what could be termed as "assay robustness". This could allow assay performance to encompass parameters such as resilience to inhibitors and/or response to changes in cycling regimes (e.g. short annealing and elongation times), which are only two among many possible examples.

#### Derivation of absolute scale via optical calibration

Although it is generally recognized that amplification efficiency can be derived from the slope of a standard curve, little or no attention has been given to the fact that the intercept establishes absolute scale by relating DNA mass to reaction fluorescence intensity [9]. Recognition of this fundamental principle presents a simple solution to the greatest limitation associated with conducting high-capacity absolute quantification using current protocols, which is reliance on target-specific standards. Based on the presumption that SYBR Green I generates similar fluorescence intensity for all amplicons, lambda gDNA can be exploited as a universal quantitative standard for establishing absolute scale, using a simple, standardized protocol referred to as optical calibration [18]. Furthermore, by relegating establishment of quantitative scale to a universal standard, error of scale is restricted to a single, well-defined entity.

Importantly, utilization of optical calibration is not limited to sigmoidal-based quantification. C_t_-based quantification can utilize the same strategy if C_t _values are converted into F_0_. This can be accomplished using the fluorescence threshold in combination with the E_max _derived from LRE analysis (equation 13). Additionally, when the fluorescence threshold is not fixed [9], the impact of inter-run differences in C_t _values are eliminated, as differences in F_t _are compensated for during the conversion of C_t _to F_0_. Finally, it should be noted that even though the principles of optical calibration described here have been developed using SYBR Green I, Swillens et al. (2004) describe an optical calibration methodology for hydrolysis probes that correlates fluorescence intensity to probe mass [34]. This suggests that probe-based assays could also implement optical calibration for conducting absolute quantification.

### Assay validation via limiting dilution assay

For any analytical technique, the ultimate performance benchmarks are accuracy and reliability. The relevancy of this to real-time qPCR is particularly evident in view of the large number of available choices for detection chemistry, enzyme formulation and instrumentation, all of which generates an enormous number of options to choose from. Indeed, many choices are based on the presumption of superior performance, even at the expense of reducing the practicalities of assay implementation and/or of increased cost. However, despite the many claims of superior performance, the paucity of supporting evidence can be striking.

Limiting dilution assay provides a fundamental, potentially universal solution to the dilemma of how to effectively assess quantitative accuracy, through the ability to conduct absolute quantification independent of real-time qPCR. LDA is simple to conduct, is independent of the kinetic and optical parameters upon which real-time qPCR is founded, does not require a quantified standard and is intrinsically self validating. As such, LDA provides potential solutions to the long-standing challenge of effectively determining true differences in assay performance, whether comparing reaction formulation, instrumentation, or as is the case for this study, data processing models. In view of the multitude of choices that currently confounds real-time qPCR, LDA could be instrumental to establishing standards in which absolute accuracy is the hallmark of assay performance.

## Conclusion

Founded upon a new paradigm for real-time qPCR, this study introduces several novel concepts and methodologies that extend the fundamental capabilities of absolute quantification. Most notable is the ability to monitor amplification kinetics within individual amplification reactions, providing the capability to reveal sample-specific inhibition that would otherwise generate unidentified quantitative errors. In addition, utilizing lambda gDNA for optical calibration not only eliminates reliance on target-specific standard curves, but as well, contributes to the standardization of real-time qPCR by centralizing the provision of quantitative scale to a single, highly defined, universal quantitative standard. Exploiting limiting dilution assay for absolute quantification provides the capability to independently evaluate absolute accuracy, irrespective of assay methodology, which could also contribute greatly to the standardization of real-time qPCR technologies. In relation to operational issues, LRE provides several attributes that facilitate large-scale absolute quantification, with the potential to extend assay performance to include the general concept of assay robustness. Ultimately, however, the ability to automate LRE quantification is most illustrative of the potential for developing high-capacity applications, reducing the resources required for conducting absolute quantification to little beyond that needed for reaction preparation.

## Methods

SigmaPlot (Version 8) was used to generate the plots presented in Figure 1 was derived from an arbitrarily selected F_C _dataset amplification profile using the SCF method [18], which produced: *k *= 1.51, *C*_1/2 _= 21.02, *F*_*max *_= 10,837, *F*_*b *_= 0. LRE analysis was conducted with MS Excel using the templates provided in additional file 2. Nonlinear correlation coefficients of predicted fluorescence profiles presented in the prototypic Java program (Figure 11) were calculated over the range of cycles encompassed by the LRE window using the equation:

$$R^{2}=1-\frac{\sum {\left(F_{C}-F_{P}\right)}^{2}}{\sum {\left(F_{C}-F_{av}\right)}^{2}}$$

where *F*_C _is reaction fluorescence and *F*_*P *_is the predicted reaction fluorescence at cycle C, with *F*_*av *_being the average reaction fluorescence generated by the cycles encompassed by the LRE window.

RNA extracted from 6-day-old Arabidopsis seedlings was reverse transcribed using oligo dT and Superscript II (Invitrogen) at a concentration of 100 ng total RNA per μl, using the manufacturer's recommended reaction conditions except that no RNase H treatment was conducted. Following 10X dilution in 10 mM Tris to a final concentration of 10 ng total RNA per μl, aliquots of the reverse transcriptase reaction were stored at -20°C.

Three different enzyme formulations were used in this study: QuantiTect (Qiagen), FullVelocity (Stratagene) and DyNAmo (Finnzymes, distributed by New England BioLabs). SYBR Green I quantity is expressed in units designated by the manufacturer (Invitrogen). SYBR Green I was diluted to the appropriate quantity using ddH_2_0 before addition to the PCR master mix just prior to amplification reaction preparation. Based upon the relative increase in F_max _as SYBR Green I quantity was increased (Figure 2), the quantity of SYBR Green I in all three commercial enzyme formulations can be estimated to be about 0.1–0.2X. The highest quantity of SYBR Green I tested in this study is thus estimated to be 10X above that typically found in commercial enzyme formulations used for real-time qPCR.

Initiated by selecting an amplicon position near to 3' end of each target transcript using the stop codon as a general landmark, primer design was primarily based upon selecting a primer length that generated a predicted T_m _= 70°C using FastPCR (Institute of Biotechnology, University of Helsinki, Finland) with parameters set to 0.05 M KCl, 0.05 M (NH_4_)_2_SO_4_, 0.0025 M MgCl_2 _and 500 nM primer concentration. Although amplicon size was restricted to 80–150 bp, no regard was given to spanning introns or to predicted secondary structures within the primers. Four amplicons were generated for each target transcript by pairing two 5' and two 3' primers. In addition to exploiting the high capacity provided by LRE analysis for assessing primer pair performance, this approach allowed quantitative accuracy to be broadly tested by the level of correlation generated from each of the four amplicons. This was particularly effective for identifying single nucleotide polymorphisms which can generate large quantitative errors produced by disrupting primer annealing to the target. Although this may not be a significant factor for highly characterized genomes, this approach can be effective for verifying quantitative accuracy for disparate species and/or new genotypes (see Results for additional details). For this study a single amplicon was selected for each target (Table 4).

**Table 4:** cDNA targets and PCR primers.

Acronym	Locus Tag	5' Primer	3' Primer	Size (bp)	Amplicon%GC
LAM	LAMCG	K7B: CTGCTGGCCGGAACTAATGAATTTATTGGT	K12: ATGCCACGATGCCTCATCACTGTTG	150	50.0%
		K23: TCTGGCTACGTCCTGATGCAGGG	K30 : TTCCTGAGACAATACAGCACGACCGCTG	150	
CHS	AT5G13930	K3: CACGACAGGAGAAGGGTTGGAGT	K4: GCGTAGGTAGGTAGGCAGATAGAAGGC	132	53.0%
SE	AT2G27100	K3: AGCTACCAAGACCTAGATGCTCCAGAG	K2: CGGGATCTCTCTAGCCCTGTCTTGT	88	48.9%
PHB	AT2G34710	K3: TCAAGCATGGGAAGGATGGTATCTTACGA	K2: AAGCTAAGCAGTGGTTTGATTCATCGTCTTCA	85	45.9%
EMF2	AT5G51230	K3: GGAGAGTGTTTATGGTGAAACTGTGGAACC	K2: GGAGCTGTTCGAGAAAGGTATTACAGTTGTTC	87	47.1%
NPR1	AT1G64280	K1: GAAATTCGTCCCTGACAGATTCGACTTCT	K4: CACTAAGAGGCAAGAGTCTCACCGAC	110	48.2%
ANT	AT4G37750	K3: CGGATCAAATATGGGCGGAAATATGAGTCCTT	K4: TCAAGAATCAGCCCAAGCAGCGAAAAC	118	48.3%
FLF/FLC	AT5G10140	K3: CACCTGCTGGACAAATCTCCGACAA	K4: TCATAATTATATATGTTTTGGATTTTGATTTCAACCGCCG	107	40.2%
HAP3C	AT2G37060	K3: GATTACGCCGCCCAGCTCAAGA	K4: GCGATGGAAGAATTTTAATTATCTGGCGAGGATTTAGG	122	45.9%
ABI3	AT3G24650	K3: CCCACCGTCGTCAGCAGCTAC	K4: TCATTTAACAGTTTGAGAAGTTGGTGAAGCGAC	115	47.0%
AP3	AT3G54340	K3: CCATGGCCTTCATGCACCCTCT	K4: ACTAAATAATTAGATAGACAATGATGGCACCAGCAAACC	110	40.0%
LEC2	AT1G28300	K3: TCCTCAATGACACCTGAGGATCACG	K4: GGGTTATGAACTTGAGAACTTCCACCACCATA	113	48.7%

Replicate amplification sets consisting of four 5.0 μl reactions in low profile tubes sealed with ultra clear caps (ABgene) were taken from a 25 μl master mix containing the target and 500 nM of each primer. For transcript quantifications, the target consisted of an aliquot of the diluted reverse transcriptase reaction containing the equivalent of 10 ng total RNA per amplification reaction, or for optical calibration, lambda gDNA (New England BioLabs) at a quantity specified in Figure 6, amplified with the lambda primers K7B-K12 (Table 4). All amplifications were conducted with a Mx3000P spectrofluorometric thermal cycler (Stratagene) using a two temperature cycling regime initiated with a 15 min activation at 95°C, followed by 50 cycles of 120 s annealing and elongation at 65°C for QuantiTect and DyNAmo or 70°C for FullVelocity and a 10 s denaturation at 95°C. To increase optical precision, three fluorescent reads were taken at the end of the annealing and elongation step and the average used as an estimate of reaction fluorescence. Specificity of amplification was confirmed by melting curve analysis conducted at the end of each run.

It is important to note that the Stratagene MxPro-Mx3000P v3.00 software used in this study was found to generate anomalous fluorescence background subtraction, as a result of including into the baseline average of 4–6 cycles after amplicon DNA first becomes detectable. This necessitated manual adjustment for each profile, of the region used to estimate baseline fluorescence. Additionally, as illustrated in Figure 10, this version of the MxPro software also attempts to correct for baseline drifting by modifying the value of each F_C _readings, a data manipulation process that cannot be disabled. This necessitated manual background subtraction as implemented in the Excel template used for LRE quantification (additional file 2). Note also that Cikos et al. (2007) reported problems with background subtraction with their Mx3000P platform which required them to conduct SCF analysis using raw fluorescence data [31].

Limiting dilution assays were conducted for each transcript target, using a master mix prepared from the reverse transcriptase reaction diluted to generate a predicted target quantity of 0.5–1 molecules per 5.0 μl amplification reaction. Dilutions were prepared in 10 mM Tris using siliconized microfuge tubes, and were based upon LRE quantification of the originating reverse transcriptase reaction. Amplifications were conducted identically to that used for LRE quantification, and the number of nil reactions scored, based upon a lack of an amplicon production. Although production of primer dimers was nearly absent across the eleven targets examined in this study, in rare cases when reactions produced primer dimers during late cycles (> 40 cycles) they were scored as negative reactions. Note that all N_0 _quantifications in this study are expressed as the number of target molecules per 10 ng total RNA.

## Authors' contributions

RGR conceived and led the project, contributed to primer design and data analysis, drafted the manuscript and wrote the Java program. DS conducted all of the real-time qPCR and LDA amplifications and much of the data analysis, in addition to maintaining the materials and primer database required by the large-scale *Arabidopsis *gene expression project from which the primers used in this study were derived.

## Supplementary Material

Additional file 1 Derivation of the two sigmoidal functions describing PCR amplification. A Microsoft Word summary of the rearrangements and substitutions used for conversion of the classic Boltzmann four parameter sigmoid function into a form in which PCR amplification can be modeled, based upon amplification dynamics as described by *ΔE *and *E*_*max *_(equation 3). Click here for file

Additional file 2 LRE data processing templates. The Excel templates used for manual data processing required for optical calibration and cDNA quantification. Click here for file

Additional file 3 LRE data summary. An Excel summary of the LRE analysis, including all of the calculations used to derive the number of molecules for eleven cDNA targets amplified using five enzyme formulations. Click here for file

Additional file 4 Prototype Java program for conducting automated LRE analysis. Java program that automates LRE quantification; requires the latest Java(TM) SE Runtime Environment available for installation at java.com. Click here for file

Additional file 5 Representative amplification profiles for each of the eleven cDNA targets. Representative F_C _datasets for the eleven cDNA targets for each of the five assay formulations. Click here for file

Additional file 6 Comparison of the Java-LRE quantification with LinReg and Miner. Excel datasheet summaries, which include all of the calculations used in the analysis for each of the three automated data processing packages. Click here for file

Additional file 7 Optical calibration amplification profiles. Excel datasheet summaries of the lambda gDNA amplification profiles used for optical calibration of the five assay formulations. Click here for file
