# Supplementary material for: A kinetic-based sigmoidal model for the polymerase chain reaction and its application to high-capacity absolute quantitative real-time PCR
Source: BMC Biotechnol. 2008 May 8;8:47. doi: 10.1186/1472-6750-8-47 (PMC2397388; doi:10.1186/1472-6750-8-47)
Supplement: Additional file 1 — Derivation of the two sigmoidal functions describing PCR amplification. A Microsoft Word summary of the rearrangements and substitutions used for conversion of the classic Boltzmann four parameter sigmoid function into a form in which PCR amplification can be modeled, based upon amplification dynamics as described by ΔE and Emax (equation 3). [file 1472-6750-8-47-S1.doc]

# Abbreviated Summary

$F_{C}=\frac{F_{\max}}{1+\text{e}^{-\left(\frac{C-C_{1/2}}{k}\right)}}+F_{b}$ **1**

$E_{C}=\frac{1+\text{e}^{-\left(\frac{C-1-C_{1/2}}{k}\right)}}{1+\text{e}^{-\left(\frac{C-C_{1/2}}{k}\right)}}-1$ **2**

$F_{\max}=\frac{E_{\max}}{-\Delta E}$ **4**

Conformity to the classic Boltzmann sigmoid function (equation 1) posses the question as to how *C_1/2_*, *k* and *F_max_* relates to *ΔE* and *E_max_*. Although equation 4 predicts that *F_max_* is defined by the ratio of *E_max_* to *ΔE*, it is less clear how *k* and *C_1/2_* relate. Indeed, a direct relationship between *E_max_* and *k* can be demonstrated by considering the scenario that if *C*=*C_1/2_*, then *E_C_*=*E_max_*/2 (Figure 1*A*) so that equation 2 becomes:

$$E_{C}=\frac{E_{\max}}{2}=\frac{1+\text{e}^{-\left(\frac{C_{1/2}-1-C_{1/2}}{k}\right)}}{1+\text{e}^{-\left(\frac{C_{1/2}-C_{1/2}}{k}\right)}}-1$$

$$E_{C}=\frac{E_{\max}}{2}=\frac{1+\text{e}^{\left(\frac{1}{k}\right)}}{1+\text{e}^{-\left(\frac{0}{k}\right)}}-1$$

$$E_{\max}=\text{e}^{\left(\frac{1}{k}\right)}-1$$

or

$k=\frac{1}{\text{Ln}(E_{\max}+1)}$ **6**

Furthermore, substituting for *k* using equation 6 and assuming *F_b_*=0, equation 1 simplifies to:

$F_{C}=\frac{F_{\max}}{1+\left(E_{\max}+1\right)^{\left(C_{1/2}-C\right)}}$ **7**

When *C*=0, *F_C_* is equal to the target quantity such that:

$F_{0}=\frac{F_{max}}{1+(E_{\max}+1)^{C_{1/2}}}$ **8**

where *F_0_* is target quantity expressed in fluorescence units. Rearrangement of equation 7 allows *C_1/2_* to be defined in terms of *F_C_*:

$C_{1/2}=\text{Log}_{(E_{\max}+1)}\left(\frac{F_{max}}{F_{C}}-1\right)+C$ **9**

so that substituting for *C_1/2_* and simplifying, equation 8 becomes:

$F_{0}=\frac{F_{\max}}{1+\left(\frac{F_{max}}{F_{C}}-1\right)\left(E_{\max}+1\right)^{C}}$ **10 (6)**

This derivative holds great significance for real-time qPCR, in that it allows fluorescence readings to be converted directly into target quantity, once estimates for *ΔE* and *E_max_* have been obtained. These derivatives can be extended one step further by defining *C_1/2_* in terms of *F_0_* by rearranging equation 8:

$C_{1/2}=\text{Log}_{(E_{\max}+1)}\left(\frac{F_{max}}{F_{0}}-1\right)$ **11**

so that substituting for *C_1/2_* and simplifying, equation 7 becomes:

$F_{C}=\frac{F_{\max}}{1+\left(\frac{F_{max}}{F_{0}}-1\right)\left(E_{\max}+1\right)^{-C}}$ **12 (7)**

In addition to allowing reaction fluorescence to be predicted for any cycle once an estimate for *F_0_* has been obtained, equation 12 also dictates that the shape of an amplification profile is determined by *ΔE* and *E_max_*, whereas profile position is determined by target quantity and *E_max_*.

# Detailed Summary

Based upon the extensive correlation that can be generated by nonlinear regression of F_C_ datasets to the classic Boltzmann four parametric sigmoid function (equation 1), it was surmised that F_max_, C_1/2_, and k could be replaced with the two kinetic parameters predicted to govern PCR amplification: ΔE and E_max_ (equation 3). Indeed equation 4 predicts that F_max_ is be derived from the ratio of E_max_ and ΔE. How then are k and C_1/2_ related to ΔE and E_max_?

$F_{C}=\frac{F_{\max}}{1+\text{e}^{-\left(\frac{C-C_{1/2}}{k}\right)}}+F_{b}$ **1**

Starting with cycle efficiency as described by equation 2:

$E_{C}=\frac{1+e^{-\left(\frac{C-1-C_{1/2}}{k}\right)}}{1+e^{-\left(\frac{C-C_{1/2}}{k}\right)}}-1$ **2**

## Relating E_max_ to k

A direct relationship between *E_max_* and *k* can be demonstrated by considering the scenario that if *C*=*C_1/2_*, then *E_C_*=*E_max_*/2 (Figure 1*A*) so that equation 2 becomes:

$$E_{C}=\frac{E_{\max}}{2}=\frac{1+\text{e}^{-\left(\frac{C_{1/2}-1-C_{1/2}}{k}\right)}}{1+\text{e}^{-\left(\frac{C_{1/2}-C_{1/2}}{k}\right)}}-1$$

$$\frac{E_{\max}}{2}=\frac{1+e^{\left(\frac{1}{k}\right)}}{1+e^{\left(\frac{0}{k}\right)}}-1=\frac{1+e^{\left(\frac{1}{k}\right)}}{2}-1$$

$$E_{\max}=2\times\left(\frac{1+e^{\left(\frac{1}{k}\right)}}{2}-1\right)=1+e^{\left(\frac{1}{k}\right)}-2$$

$$E_{\max}=e^{\left(\frac{1}{k}\right)}-1$$

$$E_{\max}+1=e^{\left(\frac{1}{k}\right)}$$

$$Ln\left(E_{\max}+1\right)=Ln\left(e^{\left(\frac{1}{k}\right)}\right)$$

$$Ln\left(E_{\max}+1\right)=\frac{1}{k}$$

$k=\frac{1}{Ln(E_{\max}+1)}$ **6**

## k substitution

Returning to the four-parametric sigmoid function, equation 1:

$F_{C}=\frac{F_{max}}{1+e^{-\left(\frac{C-C_{1/2}}{k}\right)}}-F_{b}$ **1**

Substituting for k using equation **6**:

$$F_{C}=\frac{F_{max}}{1+e^{-\left(\frac{C-C_{1/2}}{\frac{1}{Ln\left(E_{\max}+1\right)}}\right)}}$$

$$F_{C}=\frac{F_{max}}{1+e^{-\left(C-C_{1/2}\right)\times Ln\left(E_{\max}+1\right)}}$$

$$F_{C}=\frac{F_{max}}{1+\left[e^{Ln\left(E_{\max}+1\right)}\right]^{-\left(C-C_{1/2}\right)}}$$

$$F_{C}=\frac{F_{\max}}{1+\left(E_{\max}+1\right)^{-\left(C-C_{1/2}\right)}}$$

$F_{C}=\frac{F_{\max}}{1+\left(E_{\max}+1\right)^{\left(C_{1/2}-C\right)}}$ **7**

When *C*=0, *F_C_* is equal to the target quantity such that:

$$F_{0}=\frac{F_{max}}{1+(E_{\max}+1)^{\left(C_{1/2}-0\right)}}$$

$F_{0}=\frac{F_{max}}{1+(E_{\max}+1)^{C_{1/2}}}$ **8**

where *F_0_* is target quantity expressed in fluorescence units.

## Relating C_1/2_ to F_C_

Rearrangement of equation 7 allows *C_1/2_* to be defined in terms of *F_C_*:

$F_{C}=\frac{F_{\max}}{1+\left(E_{\max}+1\right)^{\left(C_{1/2}-C\right)}}$ **7**

$$\left(1+\left(E_{\max}+1\right)^{\left(C_{1/2}-C\right)}\right)\times F_{C}=F_{\max}$$

$$1+\left(E_{\max}+1\right)^{\left(C_{1/2}-C\right)}=\frac{F_{\max}}{F_{C}}$$

$$\left(E_{\max}+1\right)^{\left(C_{1/2}-C\right)}=\frac{F_{\max}}{F_{C}}-1$$

$$Log\left(E_{\max}+1\right)^{\left(C_{1/2}-C\right)}=Log\left(\frac{F_{\max}}{F_{C}}-1\right)$$

$$\left(C_{1/2}-C\right)Log\left(E_{\max}+1\right)=Log\left(\frac{F_{\max}}{F_{C}}-1\right)$$

$$\left(C_{1/2}-C\right)=\frac{Log\left(\frac{F_{\max}}{F_{C}}-1\right)}{Log\left(E_{\max}+1\right)}$$

$$\left(C_{1/2}-C\right)=Log_{\left(E_{\max}+1\right)}\left(\frac{F_{\max}}{F_{C}}-1\right)$$

$C_{1/2}=\text{Log}_{(E_{\max}+1)}\left(\frac{F_{max}}{F_{C}}-1\right)+C$ **9**

## Substituting for C_1/2_ and simplifying equation 8

$F_{0}=\frac{F_{max}}{1+(E_{\max}+1)^{C_{1/2}}}$ **8**

$$F_{0}=\frac{F_{max}}{1+(E_{\max}+1)^{\text{Log}_{(E_{\max}+1)}\left(\frac{F_{max}}{F_{C}}-1\right)+C}}$$

$F_{0}=\frac{F_{\max}}{1+\left(\frac{F_{max}}{F_{C}}-1\right)\left(E_{\max}+1\right)^{C}}$ **10 (6)**

This derivative holds great significance for real-time qPCR, in that it allows fluorescence readings to be converted directly into target quantity, once estimates for *ΔE* and *E_max_* have been obtained.

## Relating C_1/2_ to F_0_

$F_{0}=\frac{F_{\max}}{1+\left(E_{\max}+1\right)^{C_{1/2}}}$ **8**

$$1+(E_{\max}+1)^{C_{1/2}}=\frac{F_{max}}{F_{0}}$$

$$(E_{\max}+1)^{C_{1/2}}=\frac{F_{max}}{F_{0}}-1$$

$$Log(E_{\max}+1)^{C_{1/2}}=Log\left(\frac{F_{max}}{F_{0}}-1\right)$$

$$C_{1/2}Log(E_{\max}+1)=Log\left(\frac{F_{max}}{F_{0}}-1\right)$$

$$C_{1/2}=\frac{Log\left(\frac{F_{max}}{F_{0}}-1\right)}{Log(E_{\max B}+1)}$$

$C_{1/2}=Log_{(E_{\max}+1)}\left(\frac{F_{max}}{F_{0}}-1\right)$ **11**

## Substituting for C_1/2_ and simplifying equation 7

$F_{C}=\frac{F_{\max}}{1+\left(E_{\max}+1\right)^{\left(C_{1/2}-C\right)}}$ **7**

$$F_{C}=\frac{F_{\max}}{1+\left(E_{\max}+1\right)^{\left(Log_{(E_{\max}+1)}\left(\frac{F_{max}}{F_{0}}-1\right)-C\right)}}$$

$F_{C}=\frac{F_{\max}}{1+\left(\frac{F_{max}}{F_{0}}-1\right)\left(E_{\max}+1\right)^{-C}}$ **12 (7)**

In addition to allowing reaction fluorescence to be predicted for any cycle once an estimate for *F_0_* has been obtained, equation 12 also dictates that the shape of an amplification profile is determined by *ΔE* and *E_max_*, whereas profile position is determined by target quantity and *E_max_*.
